# PerioTwin-DeltaCRED: a validation-aware physiological digital twin for longitudinal periodontal treatment response prediction

**DOI:** 10.3389/fphys.2026.1958029

**Published:** 2026-09-11

**Authors:** Pradeep Kumar Yadalam

**Affiliations:** Department of Periodontics, Saveetha Dental College and Hospitals, Saveetha Institute of Medical and Technical Sciences (SIMATS), Chennai, India

**Keywords:** data re-uploading, machine learning, model calibration, periodontal digital twin, quantum machine learning, treatment response prediction

## Abstract

**Background:**

Predicting individual periodontal treatment response remains clinically unresolved. Existing machine-learning models are predominantly cross-sectional, lack a longitudinal response-vector design, and are rarely evaluated within validation-aware digital-twin frameworks that capture multidimensional credibility rather than single-endpoint discrimination alone. This study aimed to develop and evaluate PerioTwin-DeltaCRED, a validation-aware periodontal digital twin that models treatment response as a longitudinal PPD and CAL response vector, and to benchmark classical and quantum machine-learning engines against that framework.

**Methods:**

Five hundred patients with Stage II-IV periodontitis were retrospectively assembled from the Saveetha DIAS system; treatment was observational, involving either scaling and root planing alone or with low-level laser therapy. A stratified 60:20:20 split (training n=300, validation n=100, test n=100) was used for all models, using only baseline predictors—follow-up measurements and outcome variables were excluded to prevent leakage. Classical learners (logistic regression, random forest, gradient boosting) were benchmarked for poor-response classification, PPD reduction, CAL gain, and residual deep-pocket risk. Three quantum architectures—VQC-Pauli, Data Re-Uploading VQC (DRU-VQC), and QSVC—along with a hybrid quantum–classical stack, were evaluated as exploratory engines; six principal components (83.6% of variance) were angle-encoded into six-qubit circuits. Hyperparameters were optimized by Optuna on the validation set, with the test set withheld until final assessment. Uncertainty was estimated by bootstrap resampling, and the Delta-PTFI summarized credibility.

**Results:**

Classical logistic regression was the strongest poor-response model (test AUROC 0.69, 95% CI 0.59–0.80; sensitivity 0.54, specificity 0.75), with random forest comparable (AUROC 0.68) and providing the best residual deep-pocket discrimination (AUROC 0.76). Continuous responses were predicted with modest error (PPD-reduction mean absolute error 0.37 mm; CAL-gain 0.31 mm); against observed changes of 1.20 ± 0.46 mm and 0.78 ± 0.36 mm, these errors were equivalent to an intercept-only baseline. Baseline CAL and periodontal stage were the dominant and most stable predictors (mean top-5 stability 0.75), and a two-variable model using stage and baseline CAL alone matched the full model (AUROC 0.71 versus 0.69). An exploratory composite credibility index (Delta-PTFI) was 0.62, falling to 0.25 when its continuous-outcome components were anchored to an intercept-only reference. No quantum architecture surpassed classical learning: the data re-uploading classifier (DRU-VQC) was the best quantum model (test AUROC 0.63), followed by the hybrid stack (0.60), VQC-Pauli (0.46), and QSVC (0.40). Classical learners restricted to the same six principal components reached AUROC 0.58–0.66, and the best of them (random forest, 0.66) still exceeded every quantum model.

**Conclusion:**

PerioTwin-DeltaCRED provides a validation-aware digital twin for periodontal treatment response, assessing calibration, fidelity, response stability, and credibility index instead of just discrimination. Classical models outperformed, and quantum architectures like DRU-VQC showed no advantage in noiseless six-qubit tests. This is among the first studies embedding and benchmarking quantum machine learning in a periodontal digital twin; more validation and noise testing is needed before clinical use.

## Introduction

Periodontitis is a chronic inflammatory disease characterized by microbial dysbiosis with host immune dysfunction leading to periodontal breakdown and tooth loss (Sanz et al., 2020). First-line treatment includes non-surgical periodontal therapy, which can lead to clinical improvement. However, the treatment response is sometimes heterogeneous: some cases show a meaningful reduction, while others retain a residual pocket. Periodontal treatment success is measured by static clinical parameters like probing depth (PPD) and clinical attachment level (CAL) (Sanz et al., 2020). But these parameters are nonlinear, and these interactions include disease severity, treatment response and type, systemic risks, and healing response (Walter et al., 2025). Predicting periodontal response requires more than cross-sectional risk stratification. It requires a longitudinal model to understand the response better (Sarakbi et al., 2025).

Recent periodontal AI studies have demonstrated the importance of diagnosis, image-based detection of bone loss, and omics for predictive biomarkers (Kim et al., 2020; Cafiero et al., 2021). However, most studies are cross-sectional and diagnostic, and few have evaluated machine learning models to predict individual clinical response to periodontal treatment following surgical and non-surgical interventions (Khubrani et al., 2025). Periodontal AI still lacks a validation–aware physiological digital twin framework that can predict treatment response from multidimensional retrospective longitudinal data rather than from a single endpoint (Radha et al., 2023; Demuth et al., 2025) (Lee et al., 2023; Ahn et al., 2024; Bordukova et al., 2025; Sarakbi et al., 2025).

A physiological digital twin is a digital representation that models a patient’s biological condition, structure, and behaviour in a medical setting. A digital twin represents or models a patient rather than “understanding” one, and we use that terminology throughout (Lee et al., 2018; Chen et al., 2023).In the periodontal system context, the patient’s chronic inflammatory state is the physical system, and a periodontal response twin aligned with the patient’s specific vector is needed, comprising periodontal clinical parameters and variables. Classical machine learning algorithms can solve this digital twin task, especially logistic regression, and random forest can capture nonlinear interactions between staging, grading, treatment response, baseline, and clinical endpoints (PPD and CAL). These models can explain why some individuals responded poorly to treatment, and SHAP can help explain why. However, a reliable digital twin cannot be characterized by AUROC alone (Krois et al., 2019; Xue et al., 2024). It must also be calibrated, clinically interpretable, stable across resampling, and able to approximate the magnitude of PPD and CAL response. Therefore, this study evaluates classical models not only by discrimination but also by calibration, response fidelity, and clinical scenario analysis (J and TK, 2022; Gupta et al., 2025) (Felefly et al., 2023; Khubrani et al., 2025; Sadée et al., 2025; Sheza Abdul Subhan, 2025).

A quantum machine learning-based digital twin provides an additional exploratory layer for encoding periodontal clinical parameters into quantum feature spaces. This enables nonlinear data representations through rotational gates and entanglement. This study applies the data re-uploading variational quantum classifier (DRU-VQC), an architecture introduced by Pérez-Salinas et al (Roy et al., 2025), to periodontal treatment-response prediction. Data re-uploading addresses a specific limitation of standard variational quantum circuits applied to clinical tabular data: they encode the data only once at the beginning of the circuit, after which trainable layers may progressively dilute the original clinical signal (Roy et al., 2025). Repeated data re-uploading continuously reintroduces the patient’s encoded clinical features at successive circuit layers, helping the model retain feature importance while learning complex interactions. In the PerioTwin-DeltaCRED framework, DRU-VQC is evaluated as an experimental quantum architecture for modeling periodontal response patterns, with classical models serving as the main practical benchmark. The contribution claimed here is the adaptation and evaluation of this established architecture within a validation-aware periodontal digital-twin framework, not the data re-uploading method itself.

Another major need is digital twin credibility that requires verification, validation, and uncertainty quantification. This is especially important for periodontal clinical prediction, where uncalibrated models can lead to misleading patient-specific decisions. Accordingly, this study introduces PerioTwin-DeltaCRED, a validation-aware physiological digital-twin framework for longitudinal periodontal treatment-response prediction using a retrospective dataset of patients treated with SRP alone or SRP with adjunctive low-level laser therapy. The study has four objectives: model periodontal treatment response as a patient-specific longitudinal vector, benchmark classical machine-learning models for poor-response prediction, PPD reduction, CAL gain, and residual inflammatory-pocket risk; evaluate QML architectures, including VQC-Pauli, QSVC, and DRU-VQC, within a digital-twin framework; and assess model credibility via calibration, response-fidelity, explanation stability, and Delta-PTFI.

## Materials and methods

### Study design and participants

**Figure 1:**
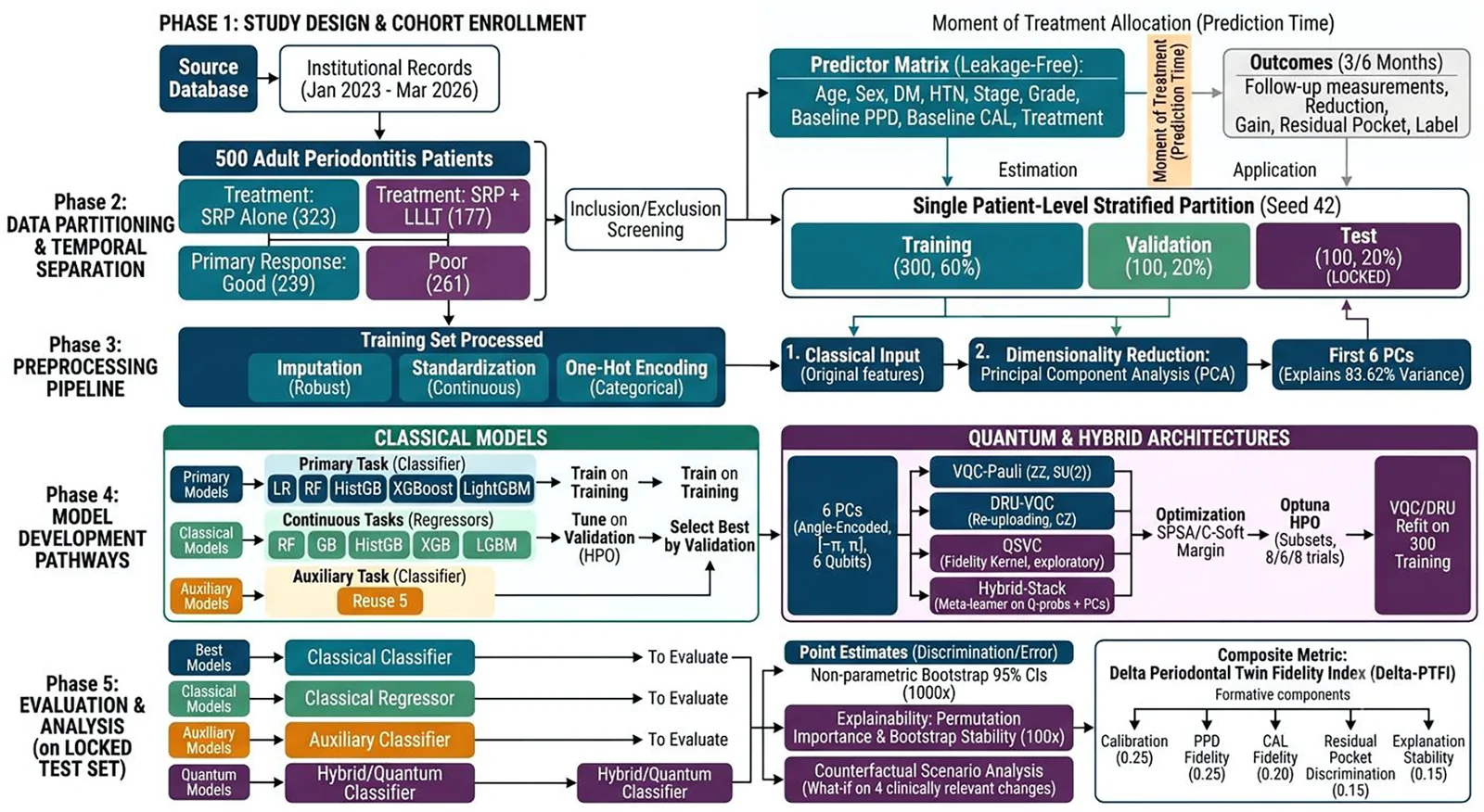
shows the workflow of the study.

This retrospective observational cohort study was conducted in the Department of Periodontics at Saveetha Dental College and Hospitals, Chennai, India. De-identified institutional clinical records of patients who received non-surgical periodontal care between January 2023 and March 2026 were retrospectively retrieved from the institutional clinical database. Data extraction, eligibility screening, de-identification, and dataset preparation for the present analysis were performed between December 2025 and March 2026. (**Figure 1**) Data retrieval and collection were performed in accordance with the Scientific Review Board-approved protocol and institutional guidelines. The three statements made elsewhere in this submission are consistent and are clarified here. The study analysed only fully de-identified clinical records generated during routine periodontal care. It involved no prospective intervention, no randomisation, no deviation from standard treatment, no additional patient contact, and no access to identifiable personal data; the analytic dataset carried no name, hospital number, or other direct or indirect identifier. The protocol was accordingly reviewed and cleared by the institutional Scientific Review Board, and under institutional policy and national guidance governing secondary analysis of anonymised routine-care records, full Institutional Ethics Committee review and individual informed consent were not required for this category of study. The submission-system entry stating that ethical approval was not required is therefore correct and is consistent with the Scientific Review Board clearance described here; the Data Availability Statement has been reworded so that all three refer to the same institutional governance process rather than appearing to name different bodies. Regarding the study timeline, the apparent inconsistency arose because March 2026 is the date on which the clinical database was queried and the analytic dataset was finalised, not the date on which the last patient was treated. Eligibility required a completed 3-month or 6-month follow-up assessment, so any patient whose follow-up window remained open at the query date was ineligible by definition; every included patient had therefore completed the full follow-up interval before extraction. A participant-flow diagram is provided as **Supplementary Table S13**.

### Study population, sample size, predictors, and data partitioning

This study retrieved 500 adult patients with Stage II-IV periodontitis who received non-surgical periodontal therapy and had complete baseline and follow-up periodontal clinical records. The sample size was determined by feasibility and model-development requirements. The final dataset included two routine-care treatment exposures: scaling and root planing alone, and scaling and root planing with adjunctive low-level laser therapy. Of the 500 included patients, 323 received SRP alone, and 177 received SRP with adjunctive LLLT. Treatment allocation was clinician-directed and retrospective, reflecting routine periodontal care rather than randomization. The primary endpoint was a clinical response after therapy. In the final dataset, 261 patients were classified as poor responders and 239 as good responders, providing balanced event representation for binary classification. A formal prediction-model sample-size assessment has now been performed following the method of Riley and colleagues rather than relying on an events-per-variable rule alone. With an outcome prevalence of 0.523, 16 candidate predictor parameters after one-hot encoding, and an anticipated Cox-Snell R-squared of 0.155 (the apparent value obtained in the development data), the criteria require approximately 848 patients for model development: 848 to keep expected shrinkage at or below 10 per cent, 286 to keep the difference between apparent and adjusted R-squared below 0.05, and 383 to estimate the overall outcome risk to within plus or minus 0.05. The available development sample of 300 patients (events per parameter 9.8) therefore falls below the most demanding of these criteria, and this is acknowledged as a principal limitation. To mitigate the resulting instability, internal validation was strengthened with repeated stratified cross-validation (10 folds repeated 20 times) on the development partition in addition to the locked test set; the two agreed closely (mean cross-validated AUROC 0.689, SD 0.090, versus 0.692 on the locked test set), indicating that the reported discrimination is not an artefact of a single fortunate split, although its precision remains limited. This study used 500 historical records: 300 to develop the model, 100 to validate the model, and 100 for locked analysis. The predictor dimensionality was restricted to factors measurable at a predetermined time; therefore, no independent test set was used for model selection and hyperparameter tuning. Treatment assignment was neither randomized nor according to a study protocol. This treatment composition (323 receiving SRP only and 177 receiving SRP and LLLT) reflects routine practice under the direction of practitioners. Each case is therefore an observational exposure.

### Inclusion criteria

Records were included if they belonged to adult patients aged ≥18 years with generalized periodontitis who were treated with SRP alone or SRP plus adjunctive low-level laser therapy and had complete baseline and follow-up periodontal measurements. Required variables included age, sex, diabetes status, hypertension status, treatment group, periodontal stage and grade, baseline mean probing depth (PPD), baseline mean Clinical attachment level (CAL), follow-up mean PPD and CAL, and final treatment response label.

### Exclusion criteria

Records were excluded if baseline or follow-up periodontal measurements were missing, the treatment-response label couldn’t be determined, or duplicates were found during quality checks. Patients with periodontal or implant surgery within the preceding 12 months, pregnancy or lactation, or immunosuppressive or chronic corticosteroid therapy were also excluded.

The primary endpoint was the documented clinical treatment-response category recorded after therapy (poor versus good response); it was not re-derived from a fixed numeric PPD/CAL threshold. Secondary outcomes included continuous PPD reduction, CAL gain, and a binary residual deep-pocket bleeding status.

### Outcome definitions

Four clinically motivated outcomes were modeled to form a patient-specific response vector. The primary outcome was a binary poor-response label, taken directly from the documented clinical response category (poor versus good response) recorded in the source clinical record; it was not re-derived from a numeric PPD/CAL threshold. Two continuous outcomes captured the magnitude of clinical improvement: PPD reduction (baseline minus follow-up mean PPD, in millimeters) and CAL gain (in millimeters). An auxiliary binary outcome, residual deep-pocket status with bleeding on probing, was a documented patient-level indicator that was positive when at least one periodontal site retained a probing depth of 5 mm or more together with bleeding on probing at the follow-up visit; a single such site was sufficient for a positive patient-level classification, and patients with no qualifying site were classified as negative. Periodontal outcomes were re-assessed at 3 months (360 patients) or 6 months (140 patients) after therapy. All four outcomes were derived exclusively from follow-up assessments and were never used as model inputs. The poor vs good classification scheme remained as the endpoint recorded in the medical record as it was used in advance as the prespecified outcome measure for recruitment and modeling. A different classification scheme, defined retrospectively with a particular PPD or CAL threshold, could not be used instead because it would constitute a redefinition of the outcome following model training. Thus, PPD decrease, CAL increase, and deep pocket presence were quantitatively examined as separate replicable endpoints. We labeled responses as poor or good based on the treatment response documented in the follow-up clinical note rather than on a specific threshold for PPD reduction or CAL gain, because no additional standardized adjudication process was available. Because this was retrospective patient information from practice, we did not independently assess inter-rater reliability for any additional rating by another clinician. Therefore, the label is best considered an outcome measure, while PPD reduction and CAL gain are secondary measures. The primary end point was the documented patient response good vs. poor. This was documented as such and not generated by application of any numeric cut-off of PPD or CAL. There was no decision rules standardized in the dataset with regard to each label nor was there any assessment IDs or duplicates included which might allow interclinician reliability to be assessed. Hence, this end point needs to be interpreted as being a pragmatic end point rather than one that defines treatment success.

Because this was done under routine conditions, we cannot discount knowledge of the treatment by the assessors. It is acknowledged that potential bias and differences between the clinicians’ classifications can exist. No measure of Cohen’s kappa or other such statistic for inter rater reliability is provided because of lack of any duplicate measures in the retrospective dataset.

### Data partitioning strategy

We generated a single patient-level stratified partition before any preprocessing and held it fixed for every model to ensure all comparisons were based on the same patients. We divided the 500 patients into training, validation, and test sets in a 60:20:20 ratio, yielding 300, 100, and 100 patients, respectively. We stratified by the poor-response label to preserve outcome prevalence across the three sets (52.3% in training, 52.0% in validation, 52.0% in test). The training set was used for preprocessing estimation and model fitting; the validation set was reserved for model and hyperparameter selection; and the test set was locked and accessed only once, after all modeling decisions had been finalized. Partitioning used a fixed random seed (42) for exact reproducibility, and the three sets were verified to be mutually exclusive at the patient level.

### Prediction time, predictor eligibility, and leakage prevention

The clinically meaningful moment of prediction was defined as immediately after treatment allocation and before any follow-up periodontal assessment. Predictor eligibility was therefore restricted to information available at or before that moment. The candidate predictors comprised age, sex, diabetes status, hypertension status, periodontal stage, periodontal grade, baseline mean PPD, baseline mean CAL, and the assigned treatment modality. All post-treatment quantities — follow-up PPD, follow-up CAL, follow-up bleeding on probing, follow-up duration, PPD reduction, CAL gain, residual deep-pocket status, and the final response label — were treated strictly as outcomes or outcome-defining variables and were excluded from the predictor matrix. This explicit temporal separation prevents target leakage and post-outcome leakage, so that reported performance reflects genuine baseline predictability rather than information from the future.

### Preprocessing and feature dimensionality reduction

**Figure 2:**
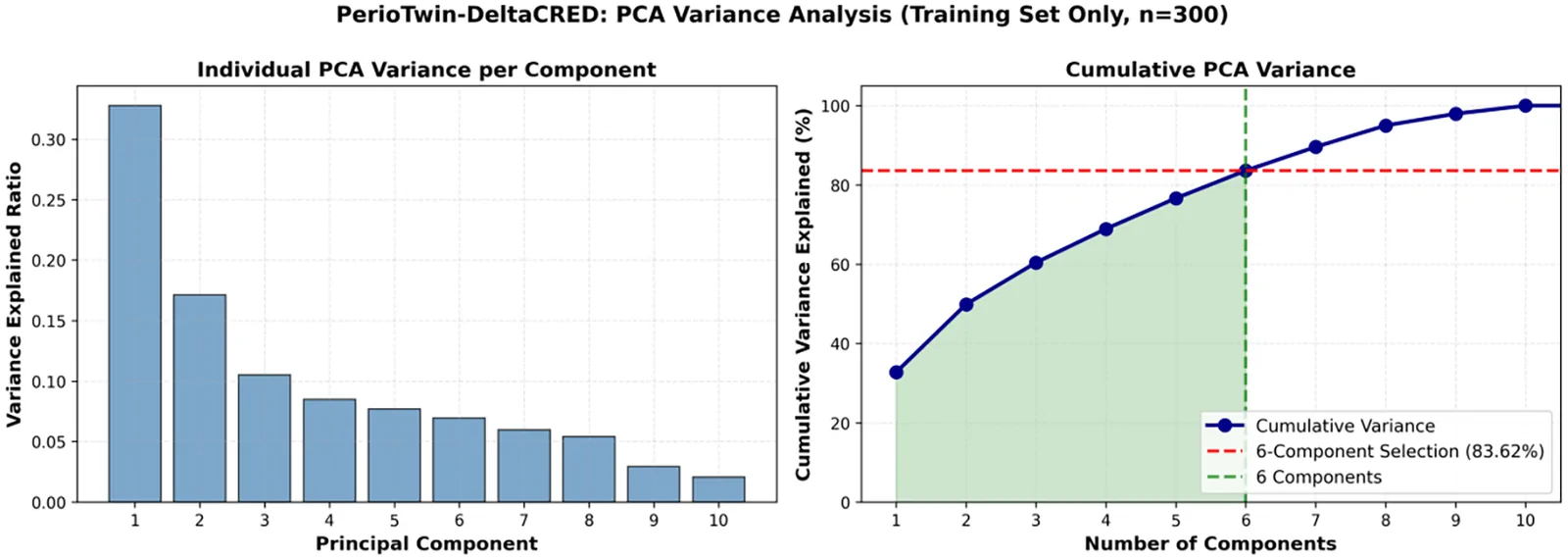
Principal component analysis fitted on the training partition (n=300). Left: variance explained by each component. Right: cumulative variance, with the six-component selection retaining 83.62% of training-set predictor variance. The fitted transformation was applied unchanged to validation and test data.

Preprocessing was implemented as a single pipeline whose parameters were estimated only on the training partition and then applied unchanged to the validation and test partitions. The analytic dataset contained no missing values in any predictor or outcome field; the pipeline nonetheless retained median imputation for continuous predictors and mode imputation for categorical predictors for robustness, but these steps were not triggered for this cohort. Continuous predictors were standardized and categorical predictors were one-hot encoded, producing a processed predictor representation used directly by the classical models. For the dimensionality-reduced representation, principal component analysis (PCA) was fitted on the processed training matrix alone; the first six principal components explained 83.62% of the training-set predictor variance (**Figure 2**), and the fitted rotation was applied unchanged to the validation and test data. Two aspects of this choice are stated transparently. First, the retention of exactly six components was determined partly by an engineering constraint, namely the six-qubit register width used for angle encoding, and not solely by a variance criterion; the six components independently retained 83.62% of training-set variance, so the choice is also defensible on statistical grounds, but it was not made on statistical grounds alone. Second, the matrix to which PCA was applied contains standardized continuous predictors together with one-hot indicator columns; this mixture affects the variance structure and the substantive interpretability of the resulting components, and the components are therefore used only as an encoding basis for the quantum circuits and for the dimensionality-matched classical comparators, and are never interpreted as clinical constructs.

### Predictive models

We evaluated five classical classifiers for the primary poor-response task: logistic regression, random forest, histogram-based gradient boosting, extreme gradient boosting (XGBoost), and LightGBM. Class weighting was applied only to logistic regression and random forest, in both cases using scikit-learn’s “balanced” scheme, which weights each class inversely to its frequency; because the primary outcome was close to balanced (52.3 per cent in the training partition), the resulting weights were near unity (approximately 0.96 and 1.05) and the scheme functioned as a safeguard rather than as a substantive correction. The gradient-boosting learners (histogram-based gradient boosting, XGBoost and LightGBM) were fitted without class weighting. For the two continuous outcomes (PPD reduction and CAL gain), we evaluated five regressors: random forest, gradient boosting, histogram-based gradient boosting, extreme gradient boosting (XGBoost), and LightGBM. The auxiliary residual deep-pocket task reused the five classification learners. The classical learners were fitted with library default hyperparameters and did not undergo any hyperparameter search; the only non-default settings were those required for convergence or reproducibility (logistic regression max_iter=1000; random forest n_estimators=300; histogram-based gradient boosting max_iter=300; XGBoost and LightGBM n_estimators=200; random_state=42 throughout). This is stated explicitly because it means the classical arm received a smaller tuning budget than the quantum arm, not a larger one. All models were trained on the training set and evaluated on the locked test set. We selected the best model for each task by validation discrimination and reported performance on the test set.

### Uncertainty quantification

Because a test set of 100 patients yields non-negligible sampling variability, point estimates were accompanied by non-parametric bootstrap confidence intervals. For the best classifier, we resampled the test set with replacement over 1000 iterations and derived 95% confidence intervals from the 2.5th and 97.5th percentiles of the bootstrap distribution for each metric. All threshold-dependent metrics (accuracy, sensitivity, specificity and F1) were computed at the default probability threshold of 0.50, which was not tuned; because the manuscript makes no claim of readiness for clinical decision making, no decision-analytic threshold optimisation or decision-curve analysis was undertaken. Bootstrap confidence intervals were derived for the selected poor-response classifier, and have now been extended to the main competing models and to the head-to-head differences between them (**Supplementary Table S9**). The patient-level predictive uncertainty referred to in an earlier version of this section was computed during development but is not reported as a result, and the claim has been removed accordingly.

### Explainability and stability

Global feature importance was quantified using permutation importance on the test set, which measures the degradation in predictive performance when each feature is randomly permuted. To assess the reliability of the resulting rankings, we performed a bootstrap stability analysis: we repeated the importance procedure across 100 training resamples and recorded the proportion of resamples in which each feature appeared among the five most important features as a stability score.

### Model-based scenario perturbation analysis

To illustrate model behavior under clinically interpretable what-if changes, we applied four model-based Scenario Perturbations to each test patient: switching SRP to SRP + LLLT, controlling diabetes, controlling hypertension, and reducing baseline PPD by 0.5 mm. For each scenario, we computed the change in predicted poor-response probability relative to the observed baseline prediction. These scenarios are model-based projections that describe the fitted model’s response surface and do not constitute causal estimates. In order to determine the reaction of the model to predefined variations in the predictors, the following four perturbations have been made in each of the patients in the testing data: Change the treatment from SRP to SRP + LLLT, change the presence of diabetes from ‘Yes’ to ‘No’, change the presence of hypertension from ‘Yes’ to ‘No’ and decrease the baseline PPD measurement by 0.5 mm. Because the treatment assignment in this situation is observational, the estimation will not focus on the average or personal causal effect. The example will show how the model’s predictions will be affected after changing the treatment, with all other measured characteristics held constant.

### Composite credibility index (Delta-PTFI)

A composite Delta Periodontal Twin Fidelity Index (Delta-PTFI) was computed to summarize several complementary dimensions of model quality on a single 0–1 scale. Each dimension was mapped to a 0–1 component score as follows: calibration = clip(1 − Brier/0.25, 0, 1), where 0.25 is the Brier score of a non-informative 0.5-probability baseline; PPD prediction fidelity = clip(1 − MAE_PPD/2.0, 0, 1) and CAL prediction fidelity = clip(1 − MAE_CAL/2.0, 0, 1), where 2.0 mm is an *a priori* clinically meaningful error ceiling; residual deep-pocket discrimination = the residual-pocket AUROC used directly; and explanation stability = the mean of the five highest bootstrap top-five inclusion proportions across 100 permutation-importance resamples. The component scores were combined using prespecified weights (0.25, 0.25, 0.20, 0.15, and 0.15, respectively): Delta-PTFI = 0.25·calibration + 0.25·PPD-fidelity + 0.20·CAL-fidelity + 0.15·residual-AUROC + 0.15·stability. The Delta-PTFI is a formative composite whose weights were assigned *a priori* based on domain reasoning rather than derived from a validation study; it is therefore reported as an exploratory summary for internal model comparison, not as a validated clinical grading scale.

### Software and reproducibility

We implemented analyses in Python using scikit-learn, XGBoost, and LightGBM for modeling, with NumPy and pandas for data handling and Matplotlib for figures. A fixed random seed (42) governed partitioning, model initialization, and bootstrap resampling. We serialized preprocessing objects and data partitions so every reported table and figure can be regenerated deterministically. Package versions were Python 3.12, scikit-learn 1.5.2, XGBoost 2.1.1, LightGBM 4.5.0, Qiskit 1.2.4 with Qiskit Aer 0.15.1, Optuna 4.0.0, NumPy 2.1.1 and pandas 2.2.3. The analysis code will be deposited in a public repository with an archived, citable release before publication.

### Quantum machine learning architectures

(J and TK, 2022; Babu et al., 2024) To examine whether near-term quantum algorithms could add predictive value beyond classical learning, four quantum and quantum-classical architectures were evaluated on the identical 60:20:20 partition and the identical leakage-free predictors. The six training-derived principal components (Section 2.5) were rescaled to the interval [-π, π] and angle-encoded onto a six-qubit register. The architectures, implemented in Qiskit, were: (i) a variational quantum classifier combining a Pauli (ZZ) feature map with a hardware-efficient SU(2) ansatz (VQC-Pauli); (ii) a data re-uploading variational classifier (DRU-VQC), implemented following the architecture of Pérez-Salinas et al (Roy et al., 2025), that re-encodes the input before every trainable block with interleaved controlled-Z entanglement; (iii) a quantum support vector classifier using a fidelity quantum kernel over a ZZ feature map (QSVC); and (iv) a hybrid model in which the class-probability outputs of the three quantum models were concatenated with the six principal components and passed to a logistic-regression meta-learner (Hybrid-Stack).

The variational classifiers (VQC-Pauli and DRU-VQC) were trained with the simultaneous perturbation stochastic approximation (SPSA) optimizer, and the quantum support vector classifier used a soft-margin regularization parameter C. Hyperparameters were selected with Optuna tree-structured Parzen estimation over 8, 6, and 8 trials for VQC-Pauli, DRU-VQC, and QSVC, respectively, maximizing validation-set AUROC; the selected configurations are reported in **Table 1**. To bound the cost of circuit optimization, this screening was performed on a balanced 80-patient training-only subset (a 50-patient subset for the QSVC kernel search). The selected VQC-Pauli and DRU-VQC configurations were then refit on the complete 300-patient training partition, so that the final variational models used all available training data. Because the fidelity kernel scales quadratically in the number of circuit evaluations, the final QSVC was trained on a balanced 120-patient subset and is reported as an exploratory kernel benchmark. The hybrid meta-learner’s inverse-regularization strength was selected by five-fold cross-validation on the training partition. The two arms did not receive identical budgets, and the difference favours the quantum arm: the classical learners were fitted with library default hyperparameters and received no hyperparameter optimization, whereas every quantum architecture received a dedicated Optuna search. On training data the final VQC-Pauli and DRU-VQC models used the complete 300-patient partition and were therefore fully matched to the classical learners; only the QSVC used a reduced 120-patient balanced subset. A budget-matched sensitivity analysis refitting the classical learners at the same reduced training-set sizes is reported in **Supplementary Table S7**. A fixed random seed (42) governed all partitioning, encoding, and optimization. All circuits were executed on a noiseless statevector simulator using an NVIDIA T4 GPU; the validation set governed every model and hyperparameter decision, and the locked test set was evaluated once.

**Table 1:** Best hyperparameter configurations selected by Optuna (validation-set AUROC).

Model	HPO trials	Best configuration	Best Val AUROC
VQC-Pauli	8	fm_reps=3, ans_reps=2, entanglement=linear, paulis=ZZ, maxiter=98	0.6114
DRU-VQC	6	n_layers=2, maxiter=97	0.5609
QSVC	8	fm_reps=3, C = 4.13	0.5310

The study follows TRIPOD+AI, using PROBAST+AI for bias self-assessment, RECORD/STROBE for retrospective reporting, and MINIMAR for AI transparency in supplementary methods. (**Supplementary Tables S1**-**S3**).

## Results

### Cohort characteristics and partition integrity

The analytic cohort comprised 500 patients with a balanced primary outcome (261 poor responders, 52.2%). Stratified partitioning produced training, validation, and test sets of 300, 100, and 100 patients with closely matched poor-response prevalence (52.3%, 52.0%, and 52.0%, respectively) and comparable demographic and baseline clinical profiles (**Table 2**). The three partitions were mutually exclusive at the patient level, and all preprocessing, scaling, encoding, and PCA were fitted using training data only. Periodontal stage, grade, treatment group and follow-up horizon are now reported by partition, together with standardized mean differences for the key continuous variables (**Supplementary Table S10**). Most differences were small, but baseline CAL and baseline PPD showed standardized differences of 0.32 and 0.26 between the training and test partitions, and Stage II disease was more frequent in the test partition (36 per cent) than in the training partition (24 per cent); because baseline CAL and stage are the dominant predictors, this imbalance is acknowledged as a source of variability in the test-set estimates. The 3-month and 6-month follow-up horizons were evenly distributed across partitions (71 to 73 per cent at 3 months). No follow-up or outcome-derived variable entered the predictor matrix, confirming the leakage-prevention design.

**Table 2:** Cohort characteristics overall and by partition. Stratification on the poor-response label preserved outcome prevalence and produced demographically comparable training, validation, and test sets.

Partition	Poor resp. %	Age, y (mean ± SD)	Female %	Diabetes %	Hypertension %	Baseline PPD, mm	Baseline CAL, mm
Overall (n=500)	52.2	48.1 ± 11.6	48.8	28.4	34.6	4.26 ± 0.65	5.73 ± 0.88
Training (n=300)	52.3	48.1 ± 11.8	49.7	28.7	33.3	4.27 ± 0.65	5.78 ± 0.87
Validation (n=100)	52.0	48.0 ± 10.8	48.0	28.0	36.0	4.41 ± 0.61	5.82 ± 0.85
Test (n=100)	52.0	48.4 ± 11.8	47.0	28.0	37.0	4.1 ± 0.63	5.49 ± 0.89

### Poor-response classification

On the locked test set, logistic regression achieved the highest observed AUROC among the five classifiers, with an area under the receiver operating characteristic curve (AUROC) of 0.6915, accuracy of 0.64, sensitivity of 0.5385, and specificity of 0.7500 (**Table 3**). Random forest ranked second (AUROC 0.6759), while the gradient-boosting variants trailed and showed weaker calibration as reflected in higher Brier scores. The ranking of logistic regression first is consistent with a modest, approximately linear baseline signal in the predictor set, but it is not statistically supported: the difference between logistic regression and random forest was 0.0156 (DeLong p = 0.621; paired bootstrap 95% CI -0.046 to +0.078, including zero). Logistic regression is therefore described throughout as the model with the highest observed AUROC rather than as statistically superior, and the two learners should be regarded as indistinguishable on this test set. Confidence intervals for the competing models are given in **Supplementary Table S9**.

**Table 3:** Poor-response classifier performance on the locked test set (n=100), ordered by AUROC. Logistic regression (bold) was the best-performing model.

Model	AUROC	AUPRC	Accuracy	Sensitivity	Specificity	F1	Brier
LogisticRegression	0.6915	0.6583	0.64	0.5385	0.7500	0.6087	0.2323
RandomForest	0.6759	0.6885	0.65	0.6154	0.6875	0.6465	0.2330
HistGradientBoosting	0.6334	0.6169	0.60	0.5962	0.6042	0.6078	0.3016
LightGBM	0.6306	0.6048	0.60	0.5192	0.6875	0.5745	0.2860
XGBoost	0.6198	0.6113	0.59	0.5769	0.6042	0.5941	0.3266

**Figure 3:**
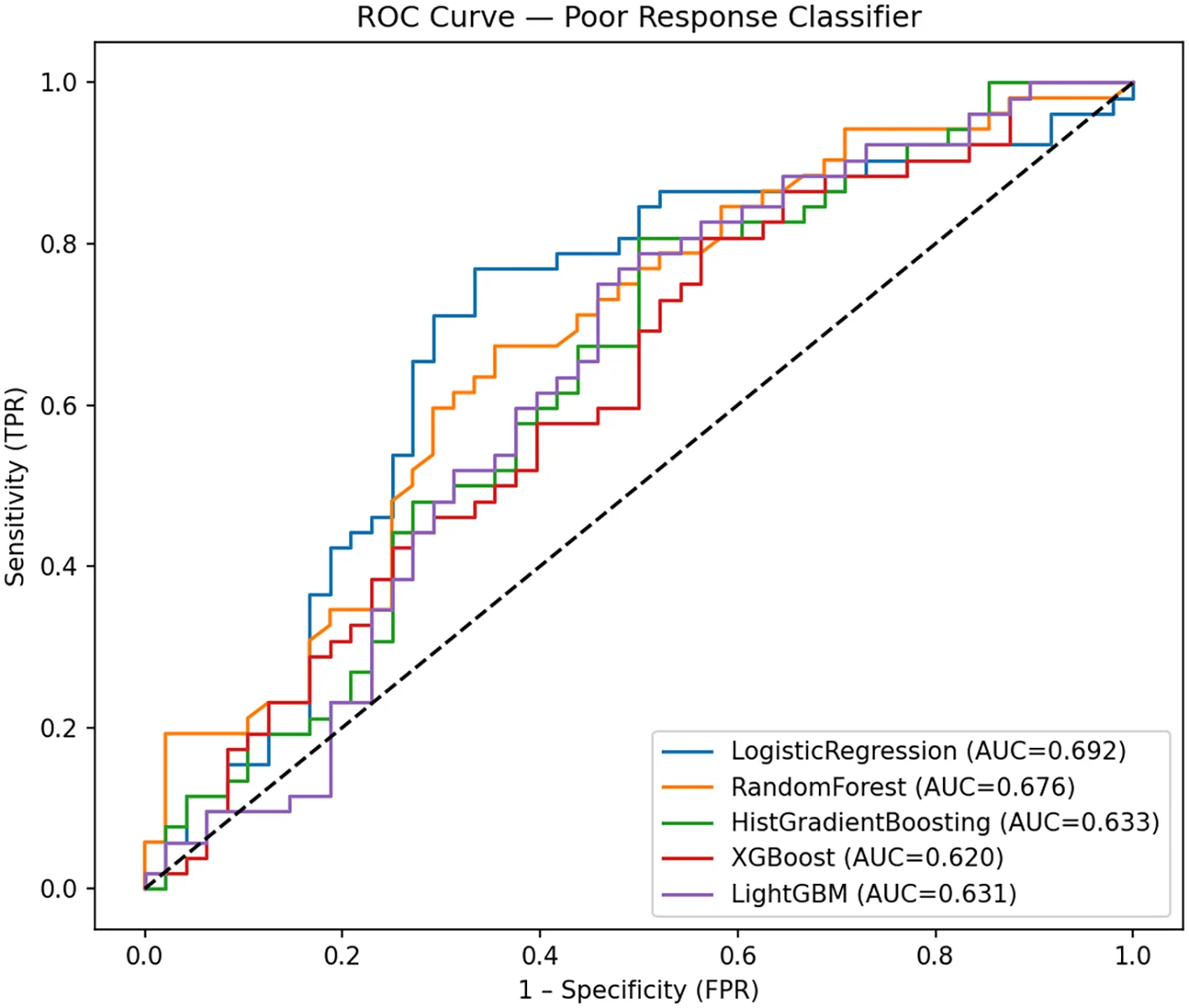
Receiver operating characteristic (ROC) curves for the five poor-response classifiers on the test set. Logistic regression achieved the highest area under the curve.

**Figure 4:**
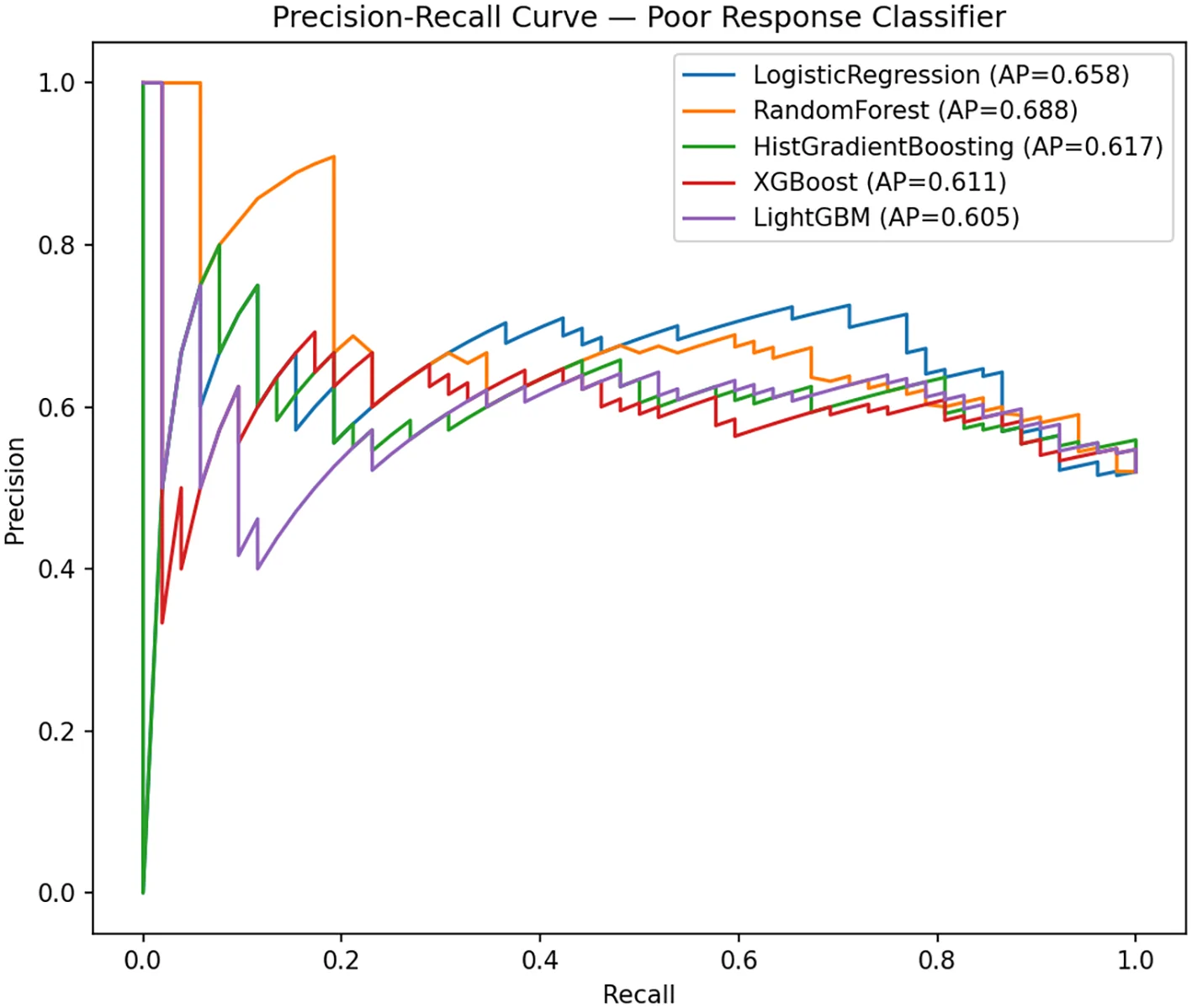
Precision–recall curves for the poor-response classifiers on the test set, summarizing the trade-off between positive predictive value and sensitivity.

The discriminative ranking of the classifiers is shown graphically in the receiver operating characteristic curves (**Figure 3**) and the precision–recall curves (**Figure 4**).

Because the test set contained 100 patients, point estimates were accompanied by 1000-iteration bootstrap 95% confidence intervals for the best classifier (**Table 4**). The AUROC interval (0.5905–0.8040) excludes 0.5, indicating discrimination beyond chance, while its width reflects the uncertainty inherent to a moderate test-set size. Sensitivity and precision showed the widest intervals, consistent with their greater sensitivity to individual misclassifications at this sample size.

**Table 4:** Bootstrap 95% confidence intervals (1000 iterations) for the best classifier (logistic regression) on the test set.

Metric	Mean	95% CI lower	95% CI upper
AUROC	0.6902	0.5905	0.8040
AUPRC	0.6720	0.5415	0.8087
Accuracy	0.6400	0.5500	0.7300
Sensitivity	0.5374	0.4042	0.6843
Specificity	0.7505	0.6200	0.8667
Precision	0.6995	0.5536	0.8334
F1-Score	0.6050	0.4857	0.7238
Brier	0.2326	0.1960	0.2710

**Figure 5:**
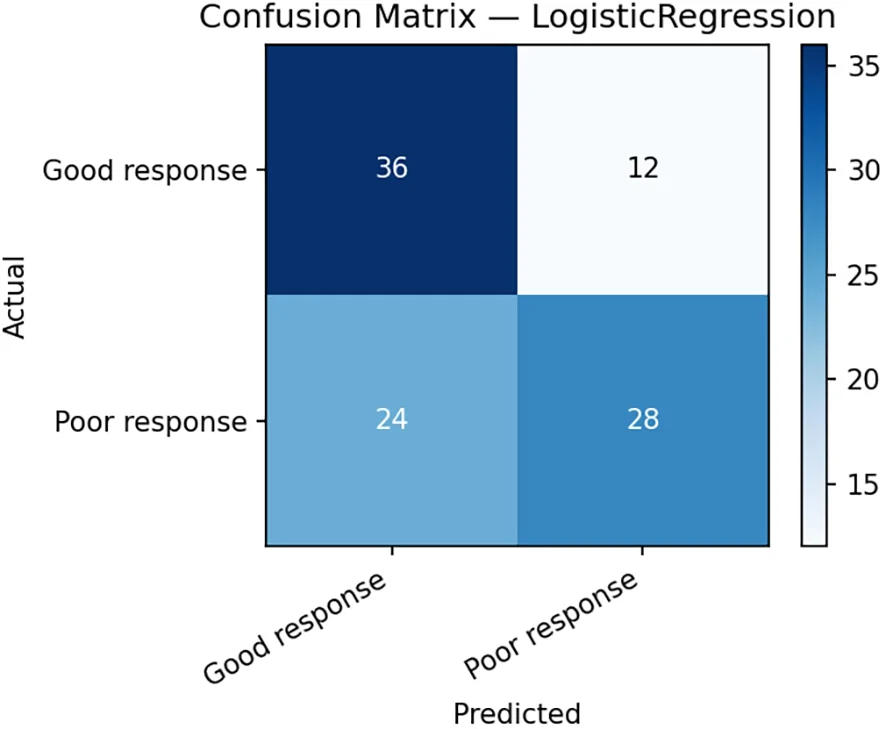
Confusion matrix for the best poor-response classifier on the test set.

**Figure 6:**
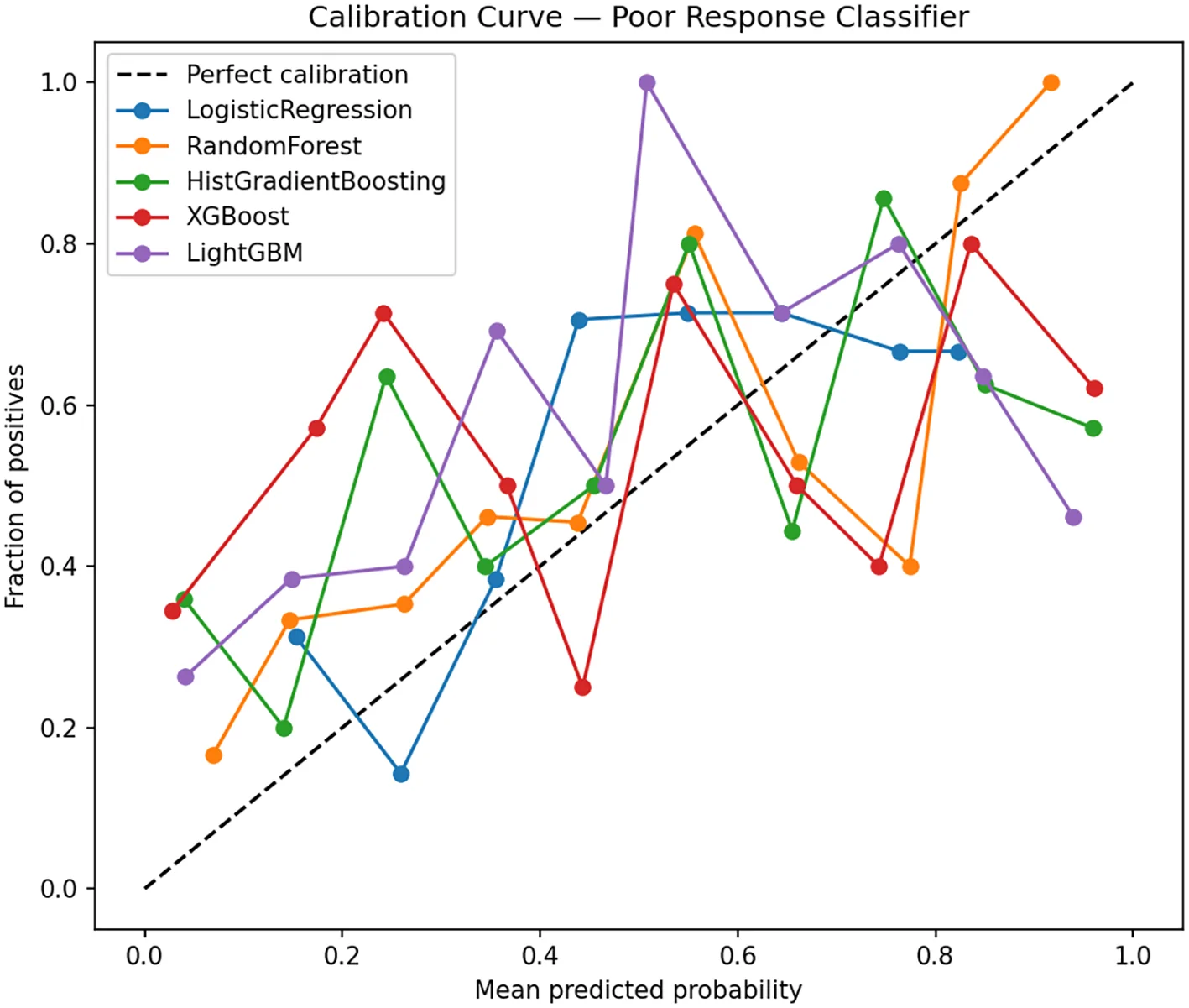
Calibration curve for the poor-response classifiers on the test set, comparing mean predicted probability with observed frequency of poor response.

The confusion matrix for the best classifier (**Figure 5**) shows a balance between correctly identified poor and good responders, and calibration was assessed formally rather than by visual inspection of the calibration curve alone (**Figure 6**). The calibration slope was 0.705 (95% CI 0.238 to 1.172) and calibration-in-the-large was 0.376 (95% CI -0.054 to 0.806); both intervals include their ideal values of 1 and 0, so calibration is consistent with adequacy but is imprecisely estimated at n = 100. Decomposing the Brier score of 0.2323 shows that only 0.0243 is attributable to miscalibration, the remainder being resolution (0.0482) and the irreducible outcome variance of a near-balanced endpoint (0.2496). The Brier score is accordingly described here as an overall measure of probabilistic accuracy rather than as a calibration statistic. Because absolute probabilities remain imprecisely calibrated, the probability changes reported in the scenario analysis should be read as relative shifts in the fitted response surface rather than as calibrated absolute risks.

### Residual deep-pocket classification

For the auxiliary task of predicting residual inflamed deep pockets, random forest achieved the highest observed AUROC (0.7582; 95% CI 0.657 to 0.853), exceeding the primary poor-response task and indicating that baseline features carry a stronger signal for persistent deep pockets than for the global response label (**Table 5**). Specificity (0.8308) exceeded sensitivity (0.4286), reflecting a conservative decision boundary at the default 0.50 threshold. As for the primary task, the margin over logistic regression (AUROC 0.7525; 95% CI 0.642 to 0.852) was not statistically supported (difference 0.0057; DeLong p = 0.869; paired bootstrap 95% CI -0.062 to +0.076), and the two models are best regarded as equivalent for this endpoint.

**Table 5:** Residual deep-pocket classifier performance on the test set (n=100), ordered by AUROC.

Model	AUROC	AUPRC	Accuracy	Sensitivity	Specificity	F1
RandomForest	0.7582	0.6080	0.69	0.4286	0.8308	0.4918
LogisticRegression	0.7525	0.6014	0.69	0.6571	0.7077	0.5974
HistGradientBoosting	0.6888	0.4927	0.65	0.3429	0.8154	0.4068

### Delta-response regression

The two continuous outcomes were predicted with modest accuracy. For PPD reduction, random forest achieved the lowest mean absolute error (MAE 0.3662 mm; RMSE 0.4570 mm), and for CAL gain it likewise performed best (MAE 0.3089 mm; RMSE 0.3750 mm) (**Tables 6**, **7**). Coefficients of determination were close to zero, indicating that the magnitude of continuous change is only weakly predictable from baseline features at this cohort size, even though the ordinal direction was captured to a limited degree (positive Spearman correlations). For context, observed PPD reduction was 1.20 ± 0.46 mm and CAL gain 0.78 ± 0.36 mm on the test set (**Table 7A**). The best-model errors therefore corresponded to 0.80 and 0.85 of the respective outcome standard deviations, and did not improve on an intercept-only baseline (MAE 0.369 mm and 0.299 mm) (**Table 7B**), indicating that the magnitude of continuous response was not meaningfully predictable from baseline variables in this cohort.

**Table 6:** PPD-reduction regression performance on the test set, ordered by MAE.

Model	MAE (mm)	RMSE (mm)	R^2^	Spearman ρ
RandomForest	0.3662	0.4570	-0.0148	0.2174
XGBoost	0.3840	0.4780	-0.1105	0.1956
GradientBoosting	0.3853	0.4752	-0.0975	0.2610
LightGBM	0.4160	0.5071	-0.2499	0.1237
HistGradientBoosting	0.4258	0.5138	-0.2828	0.1306

**Table 7:** CAL-gain regression performance on the test set, ordered by MAE.

Model	MAE (mm)	RMSE (mm)	R^2^	Spearman ρ
RandomForest	0.3089	0.3750	-0.0776	0.0696
GradientBoosting	0.3229	0.4040	-0.2507	0.1382
XGBoost	0.3268	0.4045	-0.2539	0.0955
LightGBM	0.3293	0.3997	-0.2243	0.0764
HistGradientBoosting	0.3355	0.4145	-0.3163	0.1465

**Table 7A:** Shows the observed outcome distributions.

Outcome	Mean ± SD	Median (IQR)	Range
PPD reduction	1.20 ± 0.46 mm [1.21 ± 0.44]	1.27 (0.77–1.49)	0.31 to 2.48
CAL gain	0.78 ± 0.36 mm [0.80 ± 0.33]	0.74 (0.53–1.03)	0.09 to 1.55

**Table 7B:** Shows the error placed against those distributions.

Metric	PPD reduction	CAL gain
Best-model MAE/RMSE	0.37/0.46 mm	0.31/0.38 mm
Intercept-only (train-mean) MAE/RMSE	0.369/0.454 mm	0.299/0.363 mm
Model gain vs intercept-only	+0.7%	−3.4% (worse)
MAE as fraction of observed SD	0.80	0.85
MAE as % of mean observed change	31%	39%

**Figure 7:**
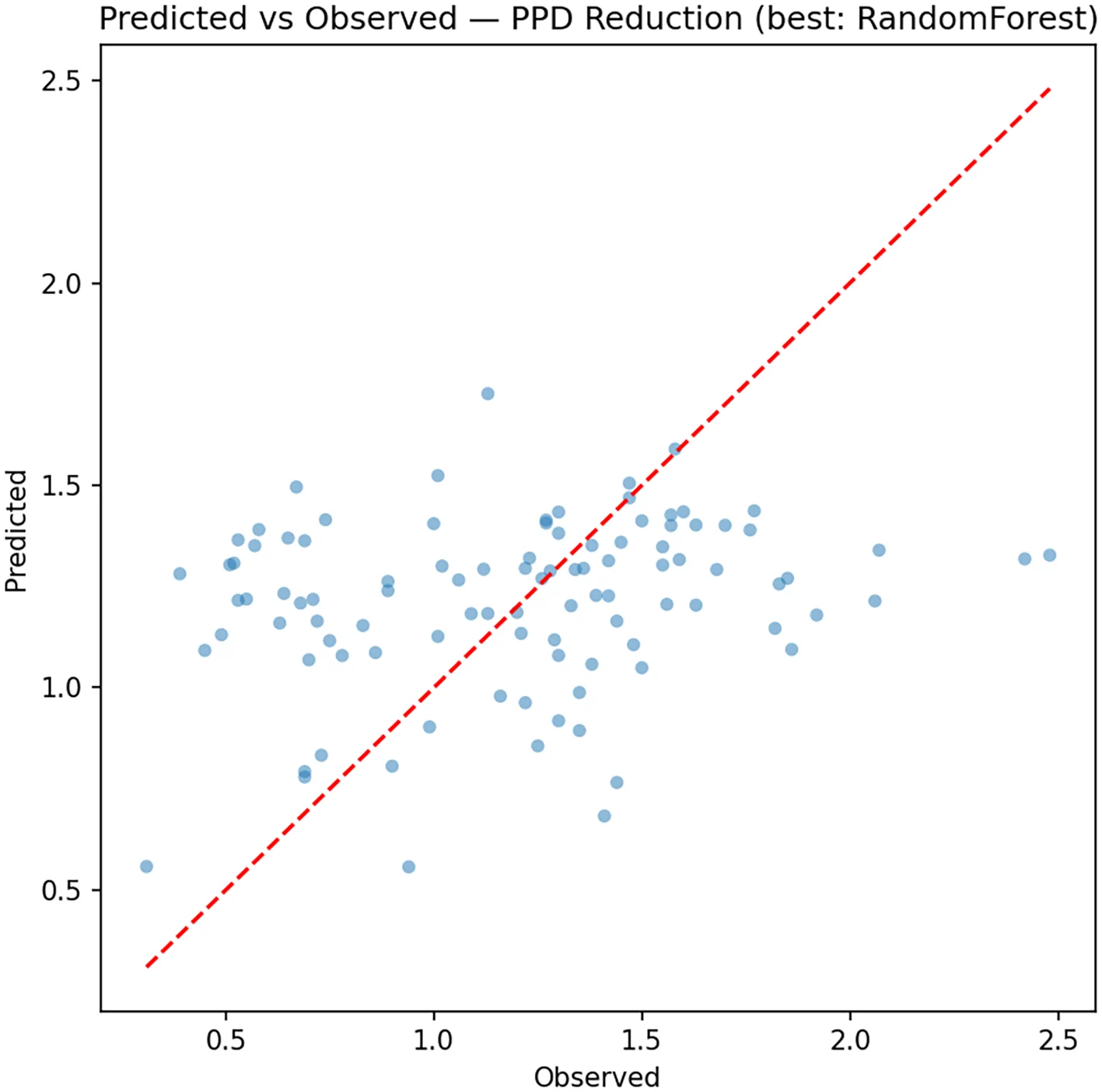
Predicted versus observed PPD reduction (mm) for the best regressor on the test set.

**Figure 8:**
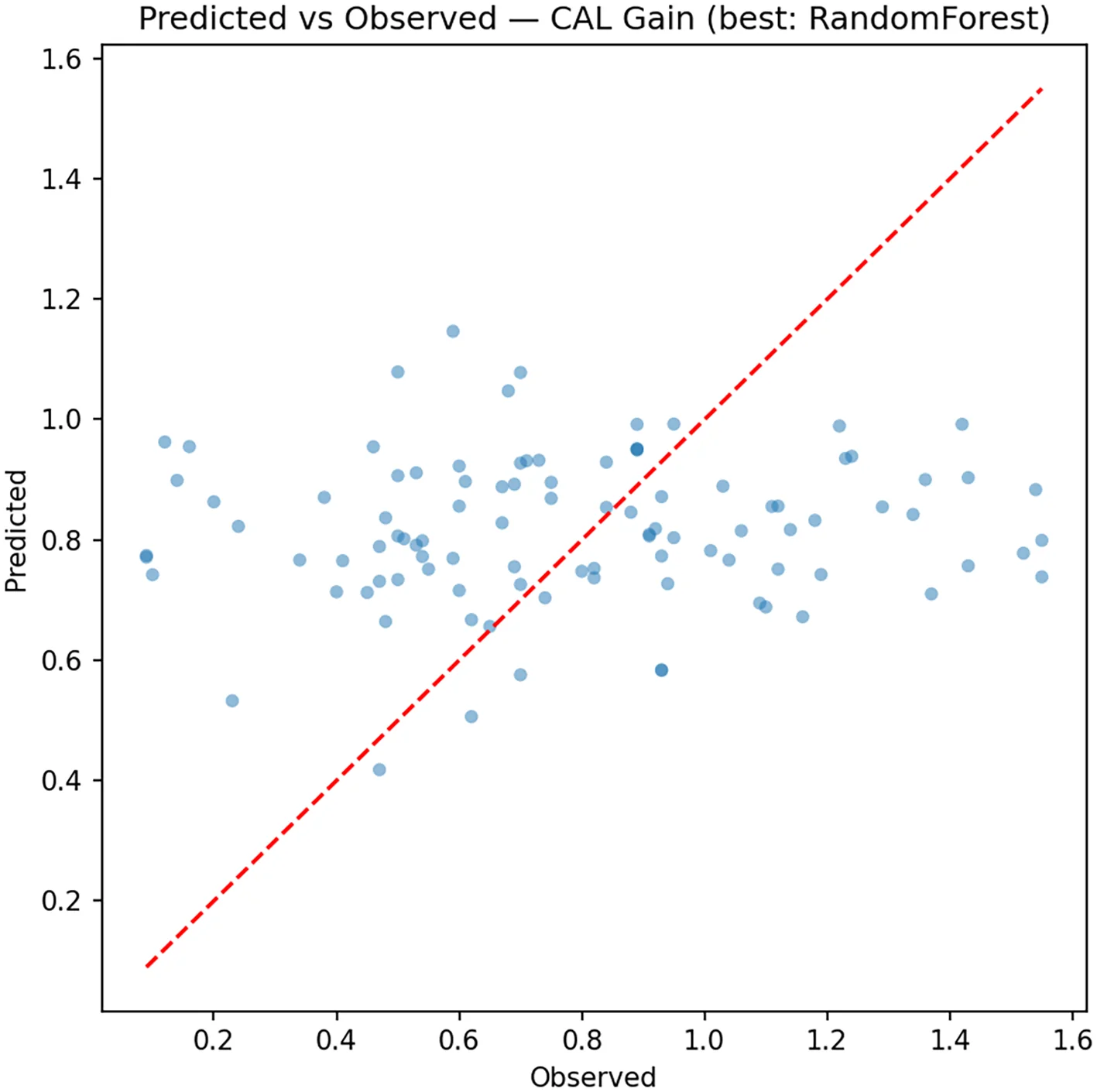
Predicted versus observed CAL gain (mm) for the best regressor on the test set.

Predicted-versus-observed relationships are shown in **Figures 7** and **8**.

### Feature importance and stability

**Figure 9:**
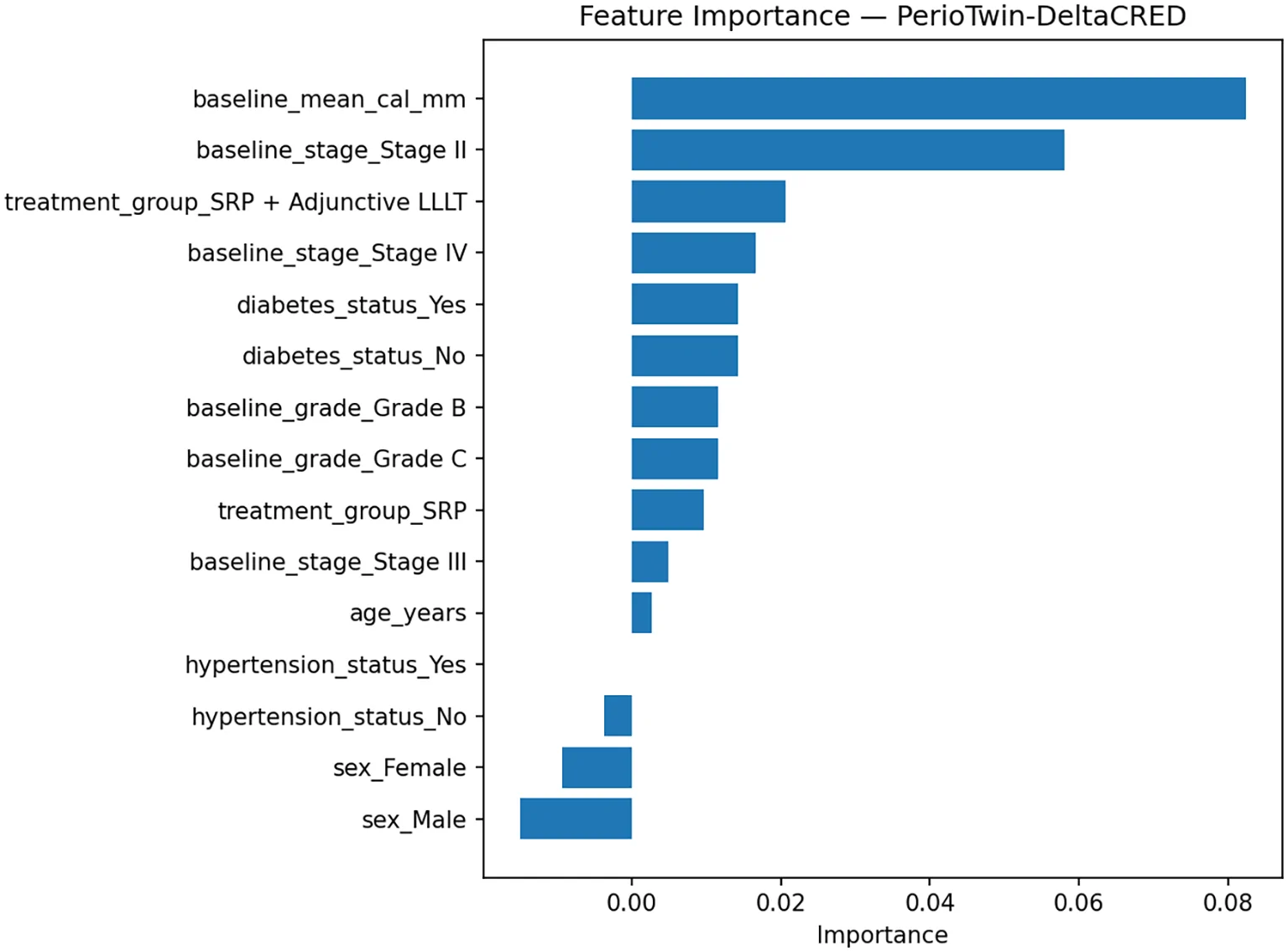
Global permutation feature importance for the best poor-response classifier.

Permutation importance identified baseline mean CAL as the single most influential predictor (importance 0.0823), followed by Stage II periodontal status and treatment modality (**Table 8**; **Figure 9**). 7Bootstrap stability analysis confirmed that these leading features were robust: baseline mean CAL appeared among the top five features in 93% of resamples and Stage II status in 92%, whereas lower-ranked features were less stable. The prominence of baseline CAL and disease stage is clinically coherent, as both index the severity of attachment loss at presentation. Because these two variables appeared to carry most of the available signal, prespecified simple clinical baselines were fitted for direct comparison with the full nine-predictor model (**Supplementary Table S11**). On the locked test set, periodontal stage alone achieved an AUROC of 0.704 (95% CI 0.607 to 0.796) and a two-variable model combining baseline CAL with stage achieved 0.714 (95% CI 0.604 to 0.813), both numerically exceeding the full model (0.692), while CAL alone achieved 0.647 (95% CI 0.535 to 0.753). None of these differences was statistically significant (DeLong p = 0.765, 0.539 and 0.373 respectively). This is an important negative finding and we state it plainly: the full nine-predictor model did not improve on a simple two-variable clinical baseline in this cohort, and a clinician using periodontal stage and baseline CAL alone would have obtained equivalent discrimination.

**Table 8:** Top ten features by permutation importance with bootstrap top-five stability (proportion of 100 resamples in which the feature ranked among the top five).

Feature	Permutation importance	Top-5 stability
baseline_mean_cal_mm	0.082333	0.93
baseline_stage_Stage II	0.058000	0.92
treatment_group_SRP + Adjunctive LLLT	0.020667	0.57
baseline_stage_Stage IV	0.016667	0.49
diabetes_status_Yes	0.014333	0.01
diabetes_status_No	0.014333	0.06
baseline_grade_Grade B	0.011667	0.05
baseline_grade_Grade C	0.011667	0.03
treatment_group_SRP	0.009667	0.46
baseline_stage_Stage III	0.005000	0.30

### Model-based scenario perturbation analysis

**Figure 10:**
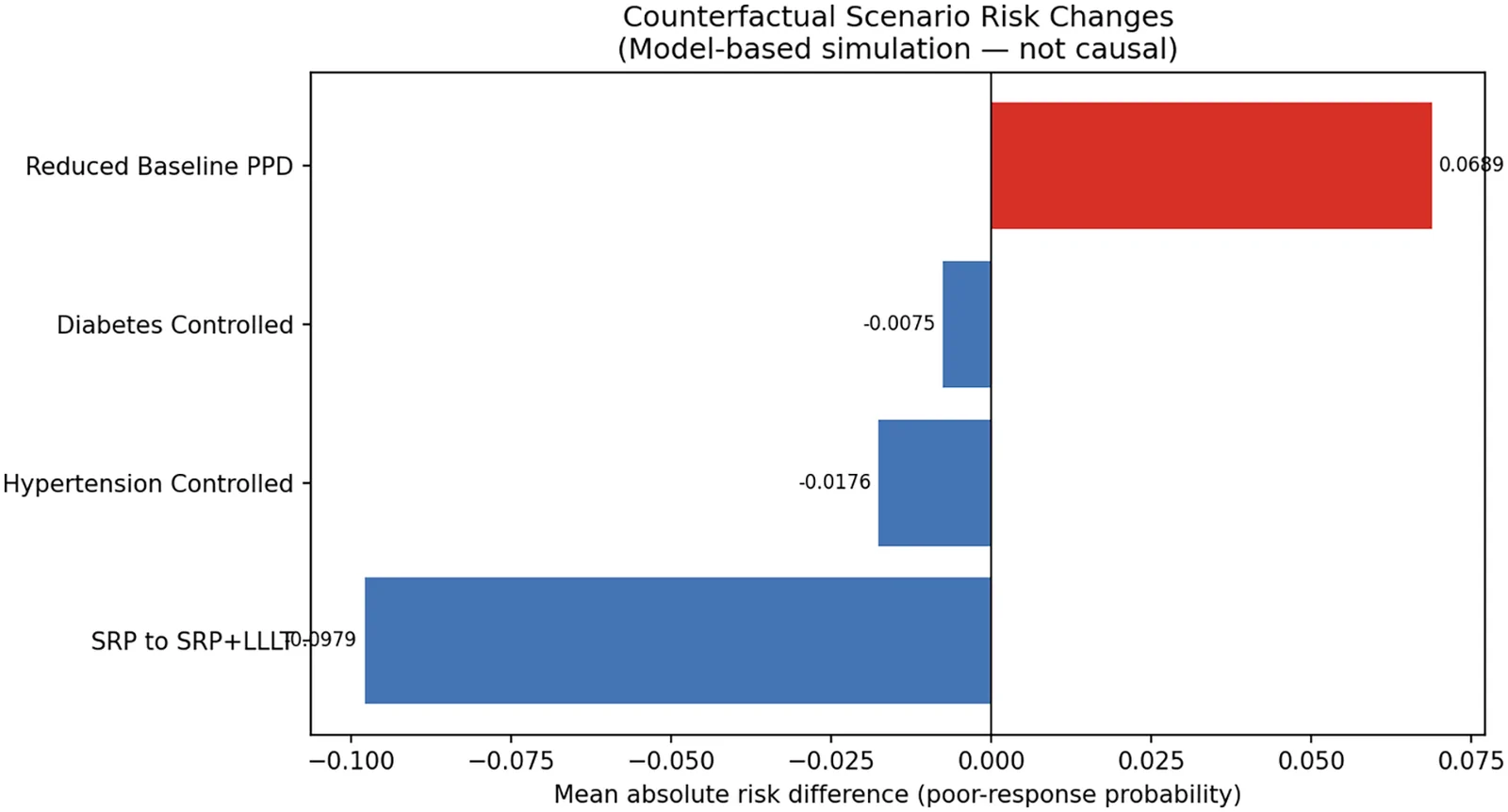
Distribution of model-based changes in predicted poor-response probability across the four Model-Based Scenario Perturbations.

Applying interpretable what-if changes to each test patient produced directionally sensible shifts in predicted poor-response risk (**Table 9**, **Figure 10**). Changing treatment from SRP to SRP+LLLT led to the largest predicted probability shift (mean Δ = −0.0979); this does not imply therapeutic benefit as treatment allocation was non-randomized. Simulated control of diabetes and hypertension yielded smaller reductions (−0.0075 and −0.0176, respectively). Reducing baseline PPD by 0.5 mm while holding baseline CAL fixed slightly increased predicted risk (+0.0689). This paradox arises because the two variables are strongly correlated in this cohort (Pearson r = 0.83, p < 0.001), so perturbing PPD alone generates clinically implausible covariate combinations in which a patient has a shallow pocket alongside unchanged severe attachment loss. When the perturbation is made correlation-consistent by reducing baseline CAL by the regression-implied 0.555 mm alongside the 0.5 mm PPD reduction, the paradox disappears entirely and the mean predicted probability is essentially unchanged (+0.0003). We therefore report the conditional perturbation as the clinically meaningful one and retain the marginal result only to illustrate the hazard of perturbing correlated clinical variables independently. These projections describe the fitted response surface and are not causal effect estimates. The analysis has been renamed a model-based scenario perturbation rather than a counterfactual analysis, and the term “risk reduction” has been avoided throughout, because the treatment exposure is observational and clinician-selected; estimating a causal treatment effect would require a defined causal estimand and an appropriate adjustment strategy, which this design does not support.

**Table 9:** Mean model-based change in predicted poor-response probability under four Model-Based Scenario Perturbations applied to all test patients (n=100).

Model-based scenario perturbation	Mean change in predicted poor-response probability
SRP to SRP+LLLT	-0.0979
Diabetes Controlled	-0.0075
Hypertension Controlled	-0.0176
Reduced Baseline PPD	+0.0689

### Composite credibility index (Delta-PTFI)

**Figure 11:**
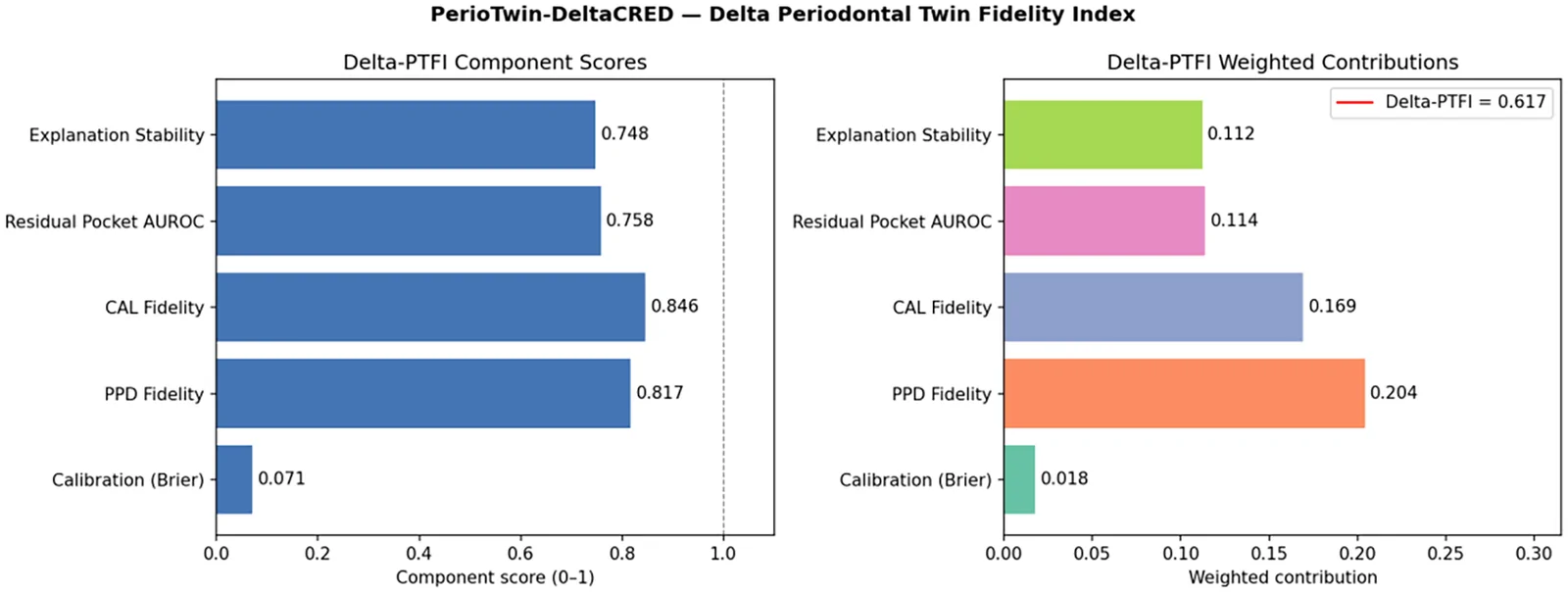
Delta-PTFI component scores (left) and their weighted contributions to the composite (right).

Integrating the five evaluation dimensions produced a Delta-PTFI of 0.6170 (**Table 10**, **Figure 11**). The composite was driven primarily by strong PPD and CAL prediction-fidelity components and by acceptable residual-pocket discrimination and explanation stability, and was limited chiefly by the calibration component. This value is, however, strongly dependent on its specification. Anchoring the PPD and CAL fidelity components to an intercept-only reference model rather than to a fixed 2.0 mm error ceiling reduces the composite from 0.617 to 0.245, because those components were scaling absolute millimetre error rather than predictive skill. Varying the error ceiling alone moves the composite between 0.387 and 0.643, and varying the component weights moves it between 0.356 and 0.798. The index also summarises the best model for each task, that is three distinct fitted models, rather than any single model. Consistent with this sensitivity and with its formative construction, the Delta-PTFI is reported as an exploratory internal summary rather than a validated grading scale, and its full re-specification, anchoring and sensitivity analyses are presented in **Supplementary Section S4** (**Supplementary Tables S4**–**S6**).

**Table 10:** Delta-PTFI component breakdown.

Component	Raw value	Component score	Weight	Weighted contribution
Calibration (Brier)	0.2323	0.0708	0.25	0.0177
PPD Fidelity	0.3662	0.8169	0.25	0.2042
CAL Fidelity	0.3089	0.8456	0.20	0.1691
Residual Pocket AUROC	0.7582	0.7582	0.15	0.1137
Explanation Stability	0.7480	0.7480	0.15	0.1122
Delta-PTFI (total)			1.00	0.6170

The composite integrates calibration, PPD fidelity, CAL fidelity, residual-pocket discrimination, and explanation stability using prespecified weights. It is an exploratory formative summary, not a validated grading scale.

### Quantum machine learning performance

On the locked test set, none of the quantum architectures surpassed the classical benchmarks. The data re-uploading classifier (DRU-VQC) was the strongest quantum-derived model (test AUROC 0.6330), followed by the hybrid quantum–classical stack (0.5954), the Pauli variational classifier (0.4611), and the quantum support vector classifier (0.3958) (**Table 11**). For reference, classical learners restricted to the same six principal components achieved test AUROC 0.58–0.66 (**Table 12**); the best of these (random forest, 0.6567) and the full-feature classical models of Section 3.2 (logistic regression, 0.6915) remained higher than every quantum model. Although DRU-VQC exceeded two of the four dimensionality-matched classical baselines, it did not surpass the best classical model. Only DRU-VQC produced a balanced decision profile (test sensitivity 0.6154, specificity 0.6250); the single-encoding VQC-Pauli (0.1923/0.8958) and the QSVC (0.9808/0.0417) collapsed toward a single class. The below-chance QSVC point estimate was verified technically: positive-class encoding was identical across all models, and every model — quantum and classical alike — was scored through the same predict_proba(…)[: 1] call, so a model-specific sign or orientation error is not possible. Confidence intervals confirm that this value is not reliably below chance: QSVC AUROC 0.396 (95% CI 0.285-0.507, p = 0.066 versus chance), VQC-Pauli 0.461 (0.348-0.575), Hybrid-Stack 0.595 (0.484-0.706), and DRU-VQC 0.633 (0.525-0.741), the last being the only quantum model whose interval excludes 0.5. The QSVC is therefore best described as indistinguishable from chance while predicting almost exclusively the positive class, a degenerate decision profile consistent with a near-uniform fidelity kernel and Platt scaling fitted on a small balanced subset. Validation-to-test differences were modest and, for DRU-VQC, in a favourable direction (0.5084→0.6330), consistent with the leakage controls in Section 2.4. On this cohort, and with noiseless six-qubit simulation, the evaluated quantum models therefore did not confer a predictive advantage over classical learning, although DRU-VQC approached the dimensionality-matched classical range. A budget-matched sensitivity analysis confirmed that this disadvantage is not an artefact of the reduced quantum training subsets (**Supplementary Table S7**): refitted on the same six principal components using the same 120-patient balanced subset on which the QSVC was trained, the classical learners still achieved test AUROC 0.634-0.645 against the QSVC value of 0.3958, and at the smaller 80-patient screening budget they achieved 0.544-0.614, still above VQC-Pauli (0.4611).

**Table 11:** Quantum and quantum-classical model performance on the locked test set (n=100).

Model	Val AUROC	Test AUROC	Sensitivity	Specificity	F1	Brier
VQC-Pauli	0.5092	0.4611	0.1923	0.8958	0.2985	0.2610
DRU-VQC	**0.5084**	**0.6330**	**0.6154**	**0.6250**	**0.6275**	**0.2438**
QSVC	0.5056	0.3958	0.9808	0.0417	0.6846	0.2722
Hybrid-Stack	0.5917	0.5954	0.4808	0.6875	0.5435	0.2633

Bold indicates the best-performing quantum-derived model on the locked test set (DRU-VQC, highest test AUROC).

**Table 12:** Classical baselines restricted to the same six principal components used by the quantum models (dimensionality-matched comparison), on the test set (n=100).

Model	5-fold CV AUROC	Val AUROC	Test AUROC	F1	Brier
XGB-PCA6	0.5598	0.5577	0.6474	0.6535	0.2986
RF-PCA6	0.5561	0.5401	0.6567	0.6327	0.2363
LR-PCA6	0.6273	0.6122	0.6546	0.5814	0.2387
GBM-PCA6	0.5327	0.5272	0.5813	0.5567	0.2824

The data re-uploading classifier DRU-VQC (bold) was the strongest quantum-derived model but remained below the best classical baselines. Final VQC-Pauli and DRU-VQC were trained on the full 300-patient training set; QSVC used a 120-patient balanced subset. The “Val AUROC” column reports post-refit validation performance, that is, the validation AUROC of the selected configuration after refitting on the full training partition. It is therefore not the same quantity as the “Best Val AUROC” of **Table 1**, which reports screening-subset performance and was the criterion used for model selection.

Hyperparameter screening used balanced training-only subsets (80 patients; 50 for the QSVC kernel search). The “Best Val AUROC” column is the best validation AUROC observed during screening and was the criterion used to select each configuration; the post-refit validation values in **Table 11** are lower because the small balanced screening subsets yield optimistically biased estimates of the selected configuration.

A multi-metric radar summary, the six-qubit PCA encoding variance, the Optuna hyperparameter-search convergence, and the hybrid meta-learner coefficients are provided in the **Supplementary Material** (**Supplementary Figures S1**–**S4**).

## Discussion

In this retrospective cohort of 500 patients, PerioTwin-DeltaCRED modeled periodontal treatment response as a longitudinal PPD–CAL response vector and evaluated it across several dimensions of digital-twin credibility, not discrimination alone. Baseline clinical variables predicted poor response with moderate discrimination; multivariable logistic regression (test AUROC 0.69, 95% CI 0.59–0.80; specificity 0.75) was strongest. Random forest was comparable (AUROC 0.68) and best for the auxiliary residual inflammatory deep-pocket endpoint (AUROC 0.76). Continuous response magnitudes showed modest error (PPD-reduction MAE 0.37 mm; CAL-gain 0.31 mm). Baseline clinical attachment level and periodontal stage were the most stable predictors (mean top-5 stability 0.75), reflecting attachment loss severity. These findings are consistent with recent work by Feher et al. (2025), who trained a random forest on baseline demographic, clinical, microbiological, and treatment-related features to predict one-year periodontal treatment response and concluded that such models require further refinement before clinical use. These results are in line with Feher et al., who found that baseline demographic, clinical, microbiological and treatment characteristics are capable of predicting periodontal treatment response in terms of random-forest modeling. However, the goal of this paper is different from trying to repeat the prediction problem solved by Feher et al. PerioTwin-DeltaCRED is aimed at predicting treatment response on several measures—poor response, PPD decrease, CAL increase and risk of inflammation pockets—and evaluating models based on calibration, response fidelity, bootstrapping variance, explanation robustness and exploratory Delta-PTFI combination index. In addition, this project involves temporal leakage prevention and benchmarking classical models against quantum and hybrid approaches with similar number of dimensions (Felefly et al., 2023; Gupta et al., 2025). A quantitative comparison clarifies where this study sits relative to the two most closely related papers. Feher et al. reported an AUROC of approximately 0.7 for one-year treatment response using a random forest trained on demographic, clinical, microbiological and treatment-related features, and Walter et al. reported comparable discrimination for step II therapy response in a larger multi-centre cohort with a guideline-anchored endpoint. Our test-set AUROC of 0.69 is therefore of the same order despite a smaller single-centre cohort and a purely clinical predictor set, but our endpoint is an institution-specific clinical label rather than a guideline-anchored one, which limits direct comparability and is the principal reason we present our estimate as a benchmark rather than as a competing model. PerioTwin-DeltaCRED reproduces this central message and extends it by quantifying predictive uncertainty through bootstrap confidence intervals, by rigorously excluding outcome-defining follow-up variables from the predictor matrix, and by summarizing credibility through the composite Delta Periodontal Twin Fidelity Index (Delta-PTFI = 0.62).In these groups, treatment is based on clinical judgement, not randomization, leading to confounding by indication and other selection biases. Therefore, group correlations and model changes don’t prove an LLLT effect (Park et al., 2023; Zeguendry et al., 2023).

Within the same framework, we evaluated three quantum architectures as exploratory computational engines. Contrary to the expectation that the data re-uploading design (Roy et al., 2025) would confer a decisive advantage, none of the quantum models surpassed the best classical learning on the leakage-free test set; the data re-uploading classifier (DRU-VQC) was nonetheless the strongest quantum-derived model (test AUROC 0.63), ahead of the hybrid quantum–classical stack (0.60), the Pauli variational classifier (0.46), and the quantum support vector classifier (0.40). When the classical learners were restricted to the identical six principal components used for six-qubit angle encoding (83.6% of retained training-set variance), they achieved AUROC 0.58–0.66; the best dimensionality-matched classical model (random forest, 0.66) narrowly exceeded DRU-VQC and the full-feature classical models remained higher still, indicating that the residual quantum disadvantage was not merely an artifact of dimensionality reduction. The data re-uploading mechanism proved far more balanced in sensitivity and specificity than the single-encoding VQC-Pauli and the QSVC, whose predictions each collapsed toward one class. These findings align with recent reviews of quantum machine learning (Felefly et al., 2023; Ahn et al., 2024; Sheza Abdul Subhan, 2025) in medicine, which remain mostly simulator-based, rely on small datasets, and lack external validation and uncertainty quantification, so reported accuracies rarely reflect true quantum advantage. By using a fixed patient partition, strict temporal leakage control, bootstrap uncertainty estimation, and a dimensionality-matched classical comparator, this study addresses these gaps. It shows that on noiseless six-qubit simulations of tabular periodontal data, quantum algorithms do not yet outperform the best classical models, although DRU-VQC narrows the gap.

The initial response was clinician-assessed and not determined through a set of criteria, quantitatively defined ahead of time. Clinicians did not agree on labels; therefore, labeling may be inconsistent, an issue to address in future studies. Several limitations qualify these conclusions. The study is single-center and retrospective; after partitioning, the effective development sample comprised 300 patients and the locked test set only 100, which explains the wide confidence intervals reported throughout and precludes strong claims of generalizability. Treatment allocation was observational and clinician-directed, so the Model-Based Scenario Perturbations reflect the model’s response surface rather than causal effects. The quantum circuits ran on a noiseless simulator, representing an optimistic bound without hardware noise. Another major limitation is missing several key risk factors like smoking, oral hygiene measures, previous periodontal maintenance, and socioeconomic status, leading to residual confounding and poorer model results. Future studies should include these predictors. The main drawback of these findings includes the absence of external validation in an independent cohort. In spite of the fact that fixed design training/validation/test provides internal validation, it does not provide the transportability of the results to other institutions, cohorts, protocols, and data recording methodologies. Consequently, these results can be considered explorative benchmarks, internally validated ones. Independent validation should be conducted prior to any clinical applications. The limitation is that the primary good/poor responder is a normal institutional clinical judgment, without an applied standard numerated criterion or assessor reliability information in the analyses, which leaves an option of inter-clinician difference and ascertainment bias (along with the awareness of follow-up treatment). The reported Area Under ROC Curve value for this criterion corresponds to the discriminatory ability of the used institution-specific label, but does not correspond to the universal state of periodontal success of treatment (Roy et al., 2025).A guideline based endpoint will be more clinically reproducible. While in the EFP Stage I – III treatment endpoint residual pockets and bleeding at site level are used, the present data set does not allow to reconstruct such a criterion due to lack of data at the site level and presence of Stage IV of the disease, in which Stage I – III criteria should be additionally qualified. No approximate *post-hoc* criterion was therefore applied. The present framework satisfies the second and third criteria and satisfies the first only in a static sense: the patient state is a fixed baseline vector, and there is no iterative state-update mechanism, no bidirectional data flow, and no mechanistic physiological process model. Practically, a simple, interpretable classical model—logistic regression—should be preferred for interpretable clinical use. The quantum approach, including the DRU-VQC, is a methodological benchmark rather than a clinical tool. Future work should involve larger, multicentre cohorts, predictor verification, external validation, calibration, noise evaluation on hardware, and consensus weighting of Delta-PTFI before quantum-enabled digital twins can be used clinically. This is among the first studies to embed and benchmark quantum machine learning within a validation-aware, longitudinal periodontal treatment-response framework.

## Conclusion

PerioTwin-DeltaCRED establishes a validation-aware physiological digital-twin framework that predicts longitudinal periodontal treatment responses, evaluating models via calibration, response fidelity, explanation stability, and a composite credibility index (Delta-PTFI), beyond just discrimination. This retrospective study used classical models as benchmarks: logistic regression had the best poor-response discrimination, and random forest had the best residual deep-pocket prediction. Quantum models, including the data re-uploading classifier of spanned AUROC 0.40–0.63; DRU-VQC was the best quantum model (0.63) but did not outperform the classical benchmarks in noiseless six-qubit simulation. This study is among the first to evaluate quantum architectures in a longitudinal periodontal treatment-response digital twin. Its leakage-controlled, uncertainty-aware design offers a reproducible benchmark, but more validation, external testing, and evaluation on noisy hardware are necessary before clinical application.

## Data Availability

The datasets analysed for this study are not publicly available because they are de-identified institutional clinical records subject to data-sharing clearance by the Scientific Review Board of Saveetha Dental College and Hospitals. Anonymised patient-level clinical variables and aggregated outcomes are available from the corresponding author on reasonable request, subject to that institutional clearance. The analysis pipeline source code, the Optuna hyperparameter-optimisation logs and the figure-generation scripts are likewise available from the corresponding author on reasonable request. No raw personal health identifiers are contained in the article, the Supplementary Material or any shared file. All aggregate results supporting the conclusions are included in the article and its **Supplementary Material**.
