# Supplementary material for: PerioTwin-DeltaCRED: a validation-aware physiological digital twin for longitudinal periodontal treatment response prediction
Source: Front Physiol. 2026 Sep 11;17:1958029. doi: 10.3389/fphys.2026.1958029 (PMC13612316; doi:10.3389/fphys.2026.1958029)
Supplement: Supplementary file 1 [file Supplementaryfile1.docx]

**Supplementary Material**

**PerioTwin-DeltaCRED: Quantum Machine Learning Diagnostics**

This Supplementary Material provides additional quantum machine-learning diagnostics referenced in the main text: a multi-metric radar comparison, the variance retained by the six-qubit PCA encoding, the Optuna hyperparameter-search convergence, the hybrid meta-learner coefficient magnitudes, and the trial-by-trial hyperparameter-optimization logs for each quantum architecture. All results derive from the identical 60:20:20 partition and leakage-free predictors used in the main analysis.

Supplementary Methods

DRU-VQC Circuit Pseudocode and Parameter Count

*Attribution. The data re-uploading architecture was introduced by Pérez-Salinas, Cervera-Lierta, Gil-Fuster and Latorre (Quantum 2020;4:226) as a universal quantum classifier. The pseudocode below describes our adaptation of that established architecture to six-qubit angle-encoded periodontal features; no claim of novelty is made for the underlying method.*

Input: x ∈ ℝ⁶ (PCA-reduced clinical features, rescaled to [−π, π]), L=2 (optimal), n=6 qubits

1. Apply H to all 6 qubits [uniform superposition: H⊗⁶]
2. For layer l = 1 to L=2:
 a. Data re-encoding: RY(xᵢ) to qubit i, for i = 0…5
 b. Trainable RY(θ₂ₗ₋₁,ᵢ) to qubit i, for i = 0…5
 c. Trainable RZ(θ₂ₗ,ᵢ) to qubit i, for i = 0…5
 d. Entanglement: CZ(i, (i+1) mod 6) for i = 0…5 [circular topology]
3. Final data re-encoding: RY (xᵢ) to qubit i, for i = 0…5
4. Measure all qubits → SamplerQNN → binary classification output

Parameter count for L=2:
 Trainable: 2 layers × 2 rotation types (RY + RZ) × 6 qubits = 24
 Data-encoding angles: 3 re-encoding passes (initial + after layer 1 + after layer 2) × 6 qubits = 18
 Total circuit parameters: 24 + 18 = 42
 Parameters optimized by SPSA: 24 (trainable only)

Optimiser: SPSA (maxiter=97; best HPO trial)

# Supplementary Figures

*Supplementary Figure S1. Radar summary of standardized test-set metrics (AUROC, AUPRC, accuracy, sensitivity, specificity, F1) for the quantum models against the classical full-feature benchmark (logistic regression). The classical benchmark forms the outermost envelope, confirming that no quantum architecture matched classical performance.*

*Supplementary Figure S2. Cumulative variance retained by the six principal components used for angle encoding onto the six-qubit register. PCA was fitted on the training partition only; the first six components retained 83.6% of the training-set predictor variance. This is the same PCA decomposition shown in main-text Figure 3, reproduced here in the quantum-encoding context.*

*Supplementary Figure S3. Optuna hyperparameter-optimization convergence (best validation AUROC achieved so far versus trial) for VQC-Pauli, DRU-VQC, and QSVC. Optimization was performed on a balanced training-only subset and never used the test set.*

*Supplementary Figure S4. Absolute logistic-regression coefficient magnitudes in the Hybrid-Stack meta-learner, showing the relative contribution of each quantum class-probability channel and each principal component.*

### Table S1. TRIPOD+AI checklist alignment

| Domain | Required reporting element | How addressed in this study |
| --- | --- | --- |
| Title/abstract | Identify study as prediction-model/AI study | Title and abstract describe PerioTwin-DeltaCRED as a clinical physiological digital twin for treatment-response prediction |
| Study design | State development, validation, or update design | Reported as a retrospective longitudinal clinical modelling study with internal validation |
| Data source | Describe source of data | Institutional de-identified periodontal records were retrieved from Saveetha Dental College and Hospitals clinical database/DIAS system |
| Participants | Define eligibility criteria | Adult patients with generalized periodontitis, SRP or SRP + adjunctive LLLT, and complete baseline/follow-up PPD and CAL were included |
| Outcome | Define target outcome clearly | Poor response was defined as failure to meet the combined criterion of ≥2 mm mean PPD reduction and ≥1 mm mean CAL gain |
| Predictors | Define all candidate predictors | Baseline demographic, systemic-risk, treatment, staging, grading, baseline PPD, baseline CAL, and prediction-horizon variables were reported |
| Leakage prevention | Exclude post-outcome variables from predictors | Follow-up PPD, follow-up CAL, PPD reduction, CAL gain, residual pocket status, and response label were excluded from predictors |
| Sample size | Explain sample size basis | 500 records were included based on feasibility and event representation; class balance supported internal model development |
| Missing data | Describe missing data handling | Records with incomplete primary outcome data were excluded; imputation was performed only within training pipelines if needed |
| Data partitioning | Report train/validation/test strategy | Patient-level stratified 60:20:20 split was used: 300 training, 100 validation, 100 test records |
| Preprocessing | Describe feature transformation | Numeric scaling, categorical one-hot encoding, and PCA for QML were fitted only on training data |
| Model development | Describe algorithms | Logistic regression, Random Forest, gradient boosting, XGBoost, VQC-Pauli, DRU-VQC, QSVC, and Hybrid-Stack were evaluated |
| Hyperparameter tuning | Describe tuning method | Validation-set-based tuning was used; QML HPO was performed using Optuna TPE on balanced subsets |
| Model validation | Describe internal validation | Final performance was reported on an independent held-out test set |
| Performance | Report discrimination and classification metrics | AUROC, AUPRC, accuracy, sensitivity, specificity, F1-score, and confusion matrix were reported |
| Calibration | Report probability calibration | Brier score and calibration curves were reported |
| Clinical usefulness | Include clinical interpretability/usefulness | SHAP explanation, residual-pocket prediction, and model-based scenario analysis were reported |
| Uncertainty | Describe uncertainty or stability | Bootstrap/explanation stability and Delta-PTFI credibility components were reported |
| Interpretability | Explain model interpretation method | SHAP-derived feature importance and top-5 stability were used |
| Generalizability | Discuss external validity | Single-centre retrospective design and need for external validation were acknowledged |
| Reproducibility | Describe software and computational environment | Python, scikit-learn, Qiskit, qiskit-machine-learning, Qiskit Aer, Optuna, and GPU infrastructure were reported |
| Transparency | Declare AI assistance and responsibility | AI-assisted writing was limited to language/clarity; authors verified all data, analysis, and conclusions |

### Table S2. PROBAST+AI risk-of-bias

| Domain | Potential risk | Mitigation in this study | Residual limitation |
| --- | --- | --- | --- |
| Participants | Single-centre institutional cohort may not represent wider periodontal populations | Consecutive eligible institutional records were used with defined inclusion/exclusion criteria | External validation is required |
| Predictors | Predictor leakage from follow-up variables | Only baseline/treatment-time predictors were used; follow-up outcomes were excluded | Follow-up duration should be interpreted as prediction horizon, not post-outcome information |
| Outcome | Composite poor-response label may combine different healing patterns | Outcome was explicitly defined using PPD and CAL thresholds; continuous PPD/CAL responses were also modelled | Alternative response definitions should be tested in future studies |
| Missing data | Retrospective records may contain incomplete fields | Records with missing primary outcome data were excluded; preprocessing pipelines handled missing predictors if present | Complete-case design can introduce selection bias |
| Model development | Overfitting from multiple algorithms and HPO | Separate training, validation, and held-out test sets were used | External validation not yet performed |
| Model evaluation | AUROC alone may overstate model utility | Calibration, Brier score, response-fidelity, residual-pocket prediction, and Delta-PTFI were added | Calibration remains limited by small test set |
| QML modelling | QML training subset was smaller than full dataset | Subset training was declared as computationally constrained exploratory benchmarking | QML findings are not evidence of clinical superiority |
| Interpretability | SHAP may be model-dependent | SHAP stability was assessed across resampling | Interpretability does not prove causality |
| Counterfactual analysis | Scenario outputs may be mistaken for causal effects | Labelled as model-based simulations, not treatment-effect estimates | Randomized or causal designs are needed for treatment-effect inference |
| Applicability | Model may not apply outside the institution | Target population and data source were explicitly defined | Multicentre temporal and external validation required |

### Table S3. RECORD/STROBE alignment for retrospective institutional clinical records

| Reporting item | How addressed |
| --- | --- |
| Data source | De-identified institutional periodontal records from Saveetha Dental College and Hospitals clinical database/DIAS system |
| Study design | Retrospective longitudinal clinical modelling study |
| Study population | Adult patients with generalized periodontitis treated with SRP or SRP + adjunctive LLLT |
| Study period | Institutional record retrieval period to be inserted |
| Eligibility | Inclusion and exclusion criteria defined before analysis |
| Data extraction | Structured tabular extraction of demographic, systemic-risk, treatment, baseline periodontal, and follow-up response variables |
| De-identification | Patient identifiers removed before computational analysis |
| Completeness | Records required complete baseline and follow-up PPD/CAL measurements |
| Bias | Retrospective single-centre design and clinician-directed treatment allocation acknowledged |
| Confounding | Treatment group interpreted as intervention-state predictor, not causal exposure |
| Validation | Patient-level train/validation/test partitioning used to prevent record-level leakage |
| Reproducibility | Model pipeline, software, and computational environment reported |
| Generalizability | Need for external validation across independent periodontal centres stated |

**Supplementary Section S4. Delta-PTFI Re-specification, Anchoring, and Sensitivity Analysis**

The Delta Periodontal Twin Fidelity Index (Delta-PTFI) is a formative composite reported as an exploratory internal summary and not as a validated clinical grading scale. Following peer review, the index has been re-specified in three respects, and its dependence on the chosen scaling constant and component weights is quantified below.

(i) Anchoring of the continuous-outcome components. In the original specification the PPD and CAL fidelity terms were computed as clip(1 - MAE/2.0, 0, 1), where 2.0 mm was an a priori clinically meaningful error ceiling. Because this constant scales absolute millimetre error rather than predictive skill, the two components have been recomputed against an empirical reference - the mean absolute error (MAE) of an intercept-only (training-mean) model evaluated on the same locked test set - as fidelity = clip(1 - MAE_model / MAE_intercept-only, 0, 1) (Table S5).

(ii) Correct labelling of the Brier-derived component. The Brier score is an overall measure of probabilistic prediction error rather than a pure calibration statistic. The transformed term is therefore renamed the overall probabilistic-accuracy component, and calibration is reported separately as the calibration slope and calibration-in-the-large with 95% confidence intervals, together with a decomposition of the Brier score into reliability, resolution, and irreducible uncertainty (Table S6).

(iii) Provenance of the composite. Delta-PTFI summarises the best model for each task - logistic regression for the probabilistic-accuracy and explanation-stability components, the random-forest regressors for PPD and CAL fidelity, and the random-forest classifier for residual deep-pocket discrimination. It therefore characterises the overall behaviour of the framework across the four modelled outcomes and is not a score for any single fitted model.

### Table S4. Sensitivity of Delta-PTFI to the error-scaling constant and to the component weights. Panel A varies only the PPD/CAL error ceiling, holding all raw component values and the prespecified weights unchanged. Panel B varies the component weights, holding the original 2.0 mm ceiling unchanged.

**Panel A. Error-scaling constant (PPD/CAL fidelity ceiling)**

| **Error ceiling (mm)** | **PPD fidelity** | **CAL fidelity** | **Delta-PTFI** |
| --- | --- | --- | --- |
| 3.00 | 0.878 | 0.897 | 0.643 |
| 2.00 (original) | 0.817 | 0.846 | 0.617 |
| 1.50 | 0.756 | 0.794 | 0.591 |
| 1.00 | 0.634 | 0.691 | 0.540 |
| 0.75 | 0.512 | 0.588 | 0.489 |
| 0.50 | 0.268 | 0.382 | 0.387 |

**Panel B. Component weighting scheme**

| **Weighting scheme** | **Delta-PTFI** |
| --- | --- |
| Prespecified (0.25 / 0.25 / 0.20 / 0.15 / 0.15) - original | 0.617 |
| Equal weights (0.20 each) | 0.648 |
| Random Dirichlet(1) weights, 20 000 draws - median | 0.675 |
| Random Dirichlet(1) weights, 20 000 draws - 95% range | 0.356 to 0.798 |

*Across defensible alternative specifications the index spans approximately half of its nominal 0-1 range (0.387 to 0.643 for the scaling constant alone; 0.356 to 0.798 across random weightings). This sensitivity is the principal reason the index is presented as an exploratory summary rather than as a headline result.*

### Table S5. Continuous-outcome fidelity components anchored to an intercept-only reference model on the locked test set (n = 100). Skill is defined as 1 - MAE_model / MAE_intercept-only; negative values are clipped to zero.

| **Continuous outcome** | **MAE, best model (mm)** | **MAE, intercept-only model (mm)** | **Skill** | **Anchored component score** |
| --- | --- | --- | --- | --- |
| PPD reduction | 0.3662 | 0.3687 | +0.007 | 0.007 |
| CAL gain | 0.3089 | 0.2987 | -0.034 | 0.000 (clipped) |

| **Specification of the continuous-outcome components** | **Delta-PTFI (total)** |
| --- | --- |
| Original (fixed 2.0 mm error ceiling) | 0.617 |
| Anchored to intercept-only model | 0.245 |

*Substituting the anchored components reduces the composite from 0.617 to 0.245. The observed test-set outcome values were 1.20 +/- 0.46 mm for PPD reduction and 0.78 +/- 0.36 mm for CAL gain; the fitted regressors therefore did not improve on a mean-only prediction, consistent with the negative coefficients of determination reported in main-text Tables 5 and 6. The high original fidelity scores (0.817 and 0.846) reflected the small absolute magnitude of periodontal change in millimetres rather than predictive skill, and should not be read as evidence that response magnitude was well predicted.*

### Table S6. Decomposition of the Brier score and candidate calibration components for the best poor-response classifier (logistic regression, test set n = 100, observed prevalence 0.52). Panel A gives the reliability-resolution-uncertainty decomposition; Panel B gives formal calibration measures; Panel C compares candidate component definitions.

**Panel A. Decomposition of the Brier score (observed Brier = 0.2323)**

| **Binning** | **Reliability (miscalibration)** | **Resolution** | **Irreducible uncertainty** | **Reconstructed Brier** |
| --- | --- | --- | --- | --- |
| 5 bins | 0.0205 | 0.0441 | 0.2496 | 0.2260 |
| 10 bins | 0.0243 | 0.0482 | 0.2496 | 0.2257 |

**Panel B. Formal calibration measures (95% confidence intervals)**

| **Measure** | **Estimate** | **95% CI** | **Ideal value** |
| --- | --- | --- | --- |
| Calibration slope | 0.705 | 0.238 to 1.172 | 1.0 |
| Calibration-in-the-large (intercept) | 0.376 | -0.054 to 0.806 | 0.0 |
| Mean predicted probability | 0.442 | not applicable | observed 0.520 |

**Panel C. Candidate definitions of the fourth component**

| **Definition** | **Value** | **What it measures** |
| --- | --- | --- |
| (a) 1 - Brier / 0.25 (original) | 0.0708 | Overall probabilistic accuracy; previously mislabelled as calibration |
| (b) 1 - Brier / [y(1-y)] (Brier skill score) | 0.0693 | Overall probabilistic accuracy, correctly labelled |
| (c) 1 - reliability / [y(1-y)] | 0.9026 | Calibration |
| (d) clip(1 - \|1 - calibration slope\|, 0, 1) | 0.7048 | Calibration |

*Interpretation. Only 0.0243 of the Brier score of 0.2323 is attributable to miscalibration; the remainder is dominated by the irreducible outcome variance of a near-balanced endpoint (0.2496). The original component score of 0.0708 therefore understated calibration quality rather than demonstrating its absence. The calibration slope (0.705) and calibration-in-the-large (0.376) have confidence intervals that include the ideal values of 1 and 0 respectively, indicating calibration that is acceptable but imprecise at n = 100. Recomputing Delta-PTFI with a calibration-specific component in place of the Brier-derived term, together with the anchored fidelity components, yields 0.453 using definition (c) or 0.404 using definition (d). Given this dependence on specification, Delta-PTFI is reported as an exploratory reporting template that requires consensus weighting and external evaluation before any comparative or clinical use.*

**Supplementary Section S5. Budget-Matched Comparison of the Classical and Quantum Arms**

The classical and quantum arms shared the same patient partition, the same leakage-free predictor source, and the same locked test set. Their training and tuning budgets were not identical, and the two asymmetries act in opposite directions. First, the classical learners were fitted with library default hyperparameters and received no hyperparameter optimization whatsoever, whereas each quantum architecture received a dedicated Optuna tree-structured Parzen search (8, 6, and 8 trials for VQC-Pauli, DRU-VQC, and QSVC respectively); this asymmetry favours the quantum arm. Second, the final VQC-Pauli and DRU-VQC models were refitted on the complete 300-patient training partition and were therefore fully matched to the classical learners on training data, whereas the QSVC alone was fitted on a reduced 120-patient balanced subset because the fidelity kernel scales quadratically in circuit evaluations.

To test directly whether the reduced training subsets could account for the observed quantum disadvantage, the classical learners were refitted on the identical six principal components at the same reduced training-set sizes and re-evaluated on the same locked test set (Table S7).

### Table S7. Budget-matched sensitivity analysis. Classical learners restricted to the same six principal components used for six-qubit angle encoding and refitted at the reduced training-set sizes used by the quantum models. All values are test-set AUROC on the same locked test set (n = 100).

| **Training set used** | **Logistic regression** | **Random forest** | **Gradient boosting** | **Quantum model at the same budget** |
| --- | --- | --- | --- | --- |
| Full 300 patients | 0.625 | 0.631 | 0.622 | DRU-VQC 0.633; VQC-Pauli 0.461 |
| 120 balanced patients (QSVC-matched) | 0.642 | 0.634 | 0.645 | QSVC 0.396 |
| 80 balanced patients (screening-matched) | 0.614 | 0.554 | 0.544 | not applicable |

*Interpretation. At the 120-patient budget on which the QSVC was trained, the classical learners still achieved test AUROC 0.634 to 0.645, compared with 0.3958 for the QSVC; the below-chance QSVC result is therefore not attributable to its smaller training subset. At the smaller 80-patient screening budget the classical learners fell to 0.544 to 0.614, still above VQC-Pauli (0.4611). Because the two variational models were already refitted on the full training partition, and because the classical models received no tuning at all, the comparison as implemented is not biased against the quantum arm. These analyses were re-run within the local pipeline; the full-training-set values (0.622 to 0.631) lie within the range reported in main-text Table 11 (0.58 to 0.66), confirming internal consistency. The reported conclusion remains a statement about the architectures, cohort size, and optimization budgets evaluated here, and the manuscript makes no claim that quantum learning is intrinsically inferior.*

**Supplementary Section S6. Verification of the QSVC Result and Uncertainty of the Quantum Model Rankings**

The QSVC test AUROC of 0.3958, with sensitivity 0.9808 and specificity 0.0417, was checked technically before being interpreted. Three checks were performed. First, the positive class was encoded identically for every model in the study (poor response = 1). Second, all models, quantum and classical alike, were scored through the same predict_proba(...)[:, 1] call and the same roc_auc_score invocation, so a sign or orientation error specific to the QSVC is not possible; had the orientation been inverted, the classical models sharing that code path would also have produced below-chance values, which they did not. Third, the QSVC was fitted with probability=True, so the score passed to the AUROC calculation is a Platt-scaled class-1 probability rather than a raw decision function.

**Table S8. Approximate 95% confidence intervals for the quantum and quantum-classical test AUROC values (Hanley and McNeil standard error; 52 poor responders and 48 good responders in the locked test set).**

| **Model** | **Test AUROC** | **95% CI** | **p vs chance** | **Interval excludes 0.5?** |
| --- | --- | --- | --- | --- |
| DRU-VQC | 0.633 | 0.525 to 0.741 | 0.016 | Yes |
| Hybrid-Stack | 0.595 | 0.484 to 0.706 | 0.092 | No |
| VQC-Pauli | 0.461 | 0.348 to 0.575 | 0.502 | No |
| QSVC | 0.396 | 0.285 to 0.507 | 0.066 | No |

*Interpretation. The confidence interval for the QSVC includes 0.5, so the apparent below-chance performance is not statistically established; the QSVC is best described as indistinguishable from chance rather than as actively anti-predictive. Its decision profile is nonetheless strongly degenerate, predicting the positive class for 98.1 per cent of test patients, which is consistent with a near-uniform fidelity kernel over the six-qubit ZZ feature map combined with Platt scaling fitted on a 120-patient balanced subset. Only DRU-VQC has an interval excluding 0.5, and the intervals of all four models overlap substantially, so the ranking among the quantum architectures should be regarded as provisional.*

**Supplementary Section S7. Relationship Between the Two Reported Validation AUROC Columns**

Two distinct validation quantities are reported for the quantum models, and they are not interchangeable. The "Best Val AUROC" column of main-text Table 12 is the best validation AUROC observed during Optuna screening, when each candidate configuration was trained on a balanced 80-patient training-only subset (50 patients for the QSVC kernel search); these are the values that were used as the model-selection criterion. The "Val AUROC" column of main-text Table 10 is the validation AUROC of the finally selected configuration after it was refitted on the complete 300-patient training partition. The screening values are higher (0.6114 versus 0.5092 for VQC-Pauli; 0.5609 versus 0.5084 for DRU-VQC; 0.5310 versus 0.5056 for QSVC) because selecting the maximum over a small balanced subset produces an optimistically biased estimate for the winning configuration. Both captions have been amended to state which quantity each column reports and which was used for selection.

**Supplementary Section S8. Uncertainty in the Head-to-Head Model Comparisons**

Model rankings on a test set of 100 patients carry substantial uncertainty. Confidence intervals are therefore reported for the main competing models, and the key head-to-head differences are assessed with both DeLong's test for two correlated ROC curves and a paired bootstrap over 2000 resamples of the test set.

### Table S9. Test-set AUROC with 95% confidence intervals for the competing classifiers, and paired uncertainty for the two key head-to-head comparisons.

| **Task and model** | **Test AUROC** | **95% CI** |
| --- | --- | --- |
| Poor response - logistic regression | 0.69 | 0.590 to 0.800 |
| Poor response - random forest | 0.68 | 0.573 to 0.784 |
| Poor response - histogram gradient boosting | 0.63 | 0.518 to 0.741 |
| Residual deep pocket - random forest | 0.76 | 0.657 to 0.853 |
| Residual deep pocket - logistic regression | 0.75 | 0.642 to 0.852 |
| Residual deep pocket - histogram gradient boosting | 0.69 | 0.581 to 0.790 |

| **Head-to-head comparison** | **Difference in AUROC** | **DeLong p** | **Paired bootstrap 95% CI** |
| --- | --- | --- | --- |
| Poor response: logistic regression minus random forest | +0.016 | 0.621 | -0.046 to +0.078 |
| Residual pocket: random forest minus logistic regression | +0.006 | 0.869 | -0.062 to +0.076 |

*Interpretation. Neither head-to-head difference is statistically supported, and both paired bootstrap intervals include zero. The manuscript therefore uses the wording "highest observed AUROC" rather than "statistically superior" for both tasks.*

**Supplementary Section S9. Balance of Key Clinical Variables Across the Data Partitions**

### Table S10. Distribution of periodontal stage, grade, treatment group and follow-up horizon across the three partitions, with standardized mean differences for the key continuous predictors.

| **Variable** | **Training (n=300)** | **Validation (n=100)** | **Test (n=100)** |
| --- | --- | --- | --- |
| Stage II / III / IV (%) | 24.0 / 57.3 / 18.7 | 22.0 / 54.0 / 24.0 | 36.0 / 45.0 / 19.0 |
| Grade B / C (%) | 53.3 / 46.7 | 47.0 / 53.0 | 51.0 / 49.0 |
| SRP / SRP + LLLT (%) | 67.0 / 33.0 | 68.0 / 32.0 | 54.0 / 46.0 |
| Follow-up 3 / 6 months (%) | 71.3 / 28.7 | 73.0 / 27.0 | 73.0 / 27.0 |

| **Continuous predictor** | **SMD training vs test** | **SMD training vs validation** |
| --- | --- | --- |
| Baseline mean CAL (mm) | +0.324 | -0.046 |
| Baseline mean PPD (mm) | +0.258 | -0.232 |
| Age (years) | -0.027 | +0.009 |

*Interpretation. Age is well balanced. Baseline CAL and baseline PPD show standardized differences of 0.32 and 0.26 between the training and test partitions, below the conventional 0.5 threshold for a moderate imbalance but not negligible, and Stage II disease is over-represented in the test partition. Because baseline CAL and stage are the dominant predictors, this contributes to the variability of the test-set estimates and is acknowledged in the Discussion. Stratification was performed on the poor-response label only, which preserved outcome prevalence but did not constrain the distribution of individual predictors.*

**Supplementary Section S10. Comparison With Simple Prespecified Clinical Baselines**

Because permutation importance indicated that baseline CAL and periodontal stage carried most of the available signal, three simple prespecified models were fitted on the training partition and evaluated on the same locked test set as the full nine-predictor model.

### Table S11. Test-set discrimination of simple clinical baselines compared with the full model (logistic regression, n = 100).

| **Model** | **Predictors** | **Test AUROC** | **95% CI** | **DeLong p vs full model** |
| --- | --- | --- | --- | --- |
| CAL alone | 1 | 0.65 | 0.535 to 0.753 | 0.373 |
| Stage alone | 2 | 0.70 | 0.607 to 0.796 | 0.765 |
| CAL + stage | 3 | 0.71 | 0.604 to 0.813 | 0.539 |
| Full model | 9 (16 parameters) | 0.69 | 0.590 to 0.800 | reference |

*Interpretation. The full nine-predictor model did not improve on a simple two-variable clinical baseline. Periodontal stage alone (0.704) and stage combined with baseline CAL (0.714) both exceeded the full model (0.692) numerically, although none of the differences reached statistical significance at this sample size. We report this as an important negative finding: on this cohort, the additional predictors contributed no measurable discrimination beyond disease stage and baseline attachment loss, and a clinician using those two variables alone would have achieved equivalent performance.*

**Supplementary Section S11. Analysis by Follow-Up Horizon**

Outcomes were assessed at 3 months in 360 patients and at 6 months in 140 patients. Because these represent different prediction horizons, baseline characteristics, outcome distributions and model performance were examined separately by horizon.

**Table S12. Baseline characteristics, outcome distributions and model discrimination by follow-up horizon.**

| **Variable** | **3 months (n=360)** | **6 months (n=140)** | **p value** |
| --- | --- | --- | --- |
| Poor responders (%) | 52.8 | 50.7 | 0.753 |
| Baseline mean PPD (mm) | 4.25 | 4.31 | not tested |
| Baseline mean CAL (mm) | 5.72 | 5.77 | not tested |
| PPD reduction (mm, mean ± SD) | 1.19 ± 0.44 | 1.25 ± 0.46 | 0.157 |
| CAL gain (mm, mean ± SD) | 0.78 ± 0.33 | 0.86 ± 0.35 | 0.019 |
| Residual deep pocket with BOP (%) | 37.5 | 33.6 | not tested |

| **Test-set subgroup** | **n** | **Poor-response AUROC** | **95% CI** |
| --- | --- | --- | --- |
| 3-month horizon only | 73 | 0.67 | 0.541 to 0.803 |
| 6-month horizon only | 27 | 0.79 | not estimated (n too small) |
| Whole locked test set | 100 | 0.69 | 0.590 to 0.800 |

*Interpretation. Baseline severity and poor-response prevalence did not differ materially by horizon, and PPD reduction was statistically indistinguishable between the two groups. CAL gain was modestly greater at 6 months (0.86 versus 0.78 mm, p = 0.019), consistent with continued attachment maturation, and this is the one outcome for which pooling is least defensible. The horizon-restricted sensitivity analysis confirms that the primary result is not driven by pooling: restricting the locked test set to the 3-month group alone yields an AUROC of 0.674 (95% CI 0.541 to 0.803), which overlaps the pooled estimate of 0.692. The two horizons were also evenly distributed across the training, validation and test partitions (71 to 73 per cent at 3 months), so the pooling does not confound the partition comparison. Follow-up interval was not available at the defined moment of prediction and was therefore correctly excluded from the predictor matrix rather than added as a baseline covariate.*

**Supplementary Section S12. Single-Seed Variational Results**

All quantum experiments used a single fixed seed (42). We acknowledge that variational classifiers trained with SPSA are sensitive to initialisation and optimiser stochasticity, and that a single run is insufficient to support a strong ranking between DRU-VQC and VQC-Pauli. Repeating the variational models across enough independent seeds to characterise run-to-run variability was not computationally feasible within the resources available for this revision: the reported quantum experiments already consumed 3433 seconds of GPU time on a Tesla T4 for a single seed, and a 10-seed replication of both variational architectures would require approximately a further two GPU-hours per architecture. We therefore state this explicitly as a limitation and have removed strong claims about differences between the quantum architectures. Consistent with this, the confidence intervals in Table S8 show that only DRU-VQC has an interval excluding 0.5 and that all four quantum intervals overlap substantially, so the ordering among quantum architectures is reported as provisional and hypothesis-generating rather than as an established ranking.

**Supplementary Section S13. Participant Flow**

### Table S13. Participant flow from the institutional database query to the final analytic cohort and its partitions.

| **Stage** | **Patients** |
| --- | --- |
| Excluded at eligibility screening () | [incomplete baseline or follow-up measurements; treatment-response label not determinable; duplicate records; periodontal or implant surgery within the preceding 12 months; pregnancy or lactation; immunosuppressive or chronic corticosteroid therapy |
| Patients meeting all inclusion criteria (analytic cohort) | 500 |
| Missing predictor or outcome values within the analytic cohort | 0 |
| Duplicate records within the analytic cohort | 0 |
| Allocated to the training partition | 300 |
| Allocated to the validation partition | 100 |
| Allocated to the locked test partition | 100 |

*Complete baseline and follow-up records were an inclusion criterion, which is why the analytic cohort contains no missing predictor or outcome values: records with missing data were removed at the eligibility-screening stage rather than being absent from the source database. This explains why the absence of missing values in 500 retrospective routine-care records, which would otherwise be unusual, is expected here.*
